# *NPEPPS* segmental duplication drives position effect expression of *TBC1D3* in the human brain

**DOI:** 10.64898/2026.01.14.699559

**Published:** 2026-01-15

**Authors:** Xavi Guitart, Jessie W Brunner, Luyao Ren, Hyeonsoo Jeong, DongAhn Yoo, David Porubsky, Kendra Hoekzema, Katherine M Munson, Kaitlyn A Sun, Marcelo Ayllon, Kaylynn Hoglin, Reed McMullen, Bryan Pavlovic, Mitchell R Vollger, Alex A Pollen, Evan E Eichler

**Affiliations:** 1. Department of Genome Sciences, University of Washington School of Medicine, Seattle, WA 98195, USA; 2. Department of Neurology, University of California, San Francisco (UCSF), San Francisco, CA 94158, USA; 3. The Eli and Edythe Broad Center of Regeneration Medicine and Stem Cell Research, UCSF, San Francisco, CA 94143, USA; 4. Howard Hughes Medical Institute, University of Washington, Seattle, WA 98195, USA; 5. European Molecular Biology Laboratory, Genome Biology Unit, 69117 Heidelberg, Germany

**Keywords:** *TBC1D3*, *NPEPPS*, segmental duplication, gene fusion, position effect, regulation, neuronal development

## Abstract

In humans, the *TBC1D3* gene family is thought to play a critical role in the expansion of the frontal cortex by promoting neuronal proliferation during brain development. This gene family shows some of the greatest structural heterozygosity (~97%) with haplotype copy numbers ranging from 3–39 among different human haplotypes. This raises the question as to how a gene so crucial in the evolutionary expansion of the human frontal cortex can be so variable in the human population. Here, we characterize the regulatory architecture that explains this paradox. We show that 45–96% of *TBC1D3* expression is attributable to a single paralog located at the most telomeric position at the edge of a cluster of *TBC1D3* genes. We find that its >3-fold higher expression relative to other copies is driven by a 110 kbp segmental duplication that occurred ~8.9 million years ago, relocating a partial duplication of the puromycin-sensitive aminopeptidase gene (*NPEPPS*), including its promoter, adjacent to this *TBC1D3* locus. Using neurospheres and comparative transcriptomics of iPSC-derived cultures, we show that expression of *NPEPPSP1-TBC1D3* increases as neurons differentiate as a result of alternative splicing and differential polyadenylation usage. While the fusion exists in other ape lineages, we show subsequent deletion of the *NPEPPSP1* promoter in *Gorilla* and a separate, lineage-specific duplication in the *Pan* lineage ablated the production of this fusion product, rendering this position effect of *TBC1D3* specific to humans.

## INTRODUCTION

*TBC1D3* is a primate-specific gene family that promotes cellular proliferation in both neurodevelopment and cancer. Originally identified in prostate and breast cancer tumors, functional experiments determined that *TBC1D3* promotes cell transformation and proliferation by manipulating cell vesicle transport, specifically amplifying the effect and duration of insulin-like growth factor (IGF) and epidermal growth factor (EGF) pathway-associated receptors (Frittoli et al., 2008, Wainszelbaum et al., 2008 & 2012). While its cell proliferation is co-opted in cancers, endogenous *TBC1D3* plays a developmental role by potentially expanding neural progenitor populations in the primate cerebral cortex, particularly in outer radial glia (Ju et al., 2016). Subcellular localization studies suggest that while *TBC1D3* is generally expressed cytoplasmically, in neuronal contexts *TBC1D3* translocates to the nucleus, where it inhibits the histone methyltransferase G9a (Hou et al., 2021). This repression reduces H3K9me2-mediated gene silencing and delays differentiation of outer radial glial cells, leading to additional division rounds and potentially contributing to the exponential increase in cortical neurons associated with human brain development.

The corresponding *TBC1D3* gene family is embedded in a dynamic core duplicon and has expanded recently during primate evolution via segmental duplications (SDs) across chromosome 17 (Jiang et al., 2007). While many of the orphan *TBC1D3* paralogs are thought to be nonfunctional pseudogenes, in humans, transcribed *TBC1D3* copies that maintain an open reading frame (ORF) originate primarily from two major clusters, Cluster 1 and Cluster 2, mapping to chromosome 17q12 (Guitart et al., 2024). These SD clusters also promote non-allelic homologous recombination leading to a recurrent 17q12 deletion/duplication (known as renal cyst and diabetes or RCAD syndrome) (Mitchel & Moreno-De-Luca et al., 1993; Mefford et al., 2007, 2016). Both the duplication and deletion syndromes manifest heterogeneously in patients, though the deletion syndrome is more often associated with abnormalities of the renal and endocrine systems. In contrast, the duplication syndrome is associated with both neurodevelopmental and psychiatric abnormalities. While 75% of deletion events are *de novo*, 90% of duplications are inherited, suggesting that the duplication may cause a less severe phenotype and thus persist in the general population for a few generations (Mitchel & Moreno-De-Luca et al., 1993; Mefford et al., 2016).

The origin and expansion of *TBC1D3* is complex: *TBC1D3* was proposed to originate from *USP6NL* (i.e., *RNTRE*)—given the conserved exon structure across 13 of 14 exons—though at the amino acid level the proteins share only 34% identity suggesting extensive amino acid replacement over a short period of primate evolution (Frittoli et al., 2008). In our prior work, we characterized the evolution and copy number diversity of the *TBC1D3* gene family among primates (Guitart et al., 2024).We showed *TBC1D3* expanded independently across seven separate lineages of the simian infraorder but is generally absent from prosimians, also known as Strepsirrhines. Within humans, *TBC1D3* is estimated to have expanded a human-specific modified version of the predicted protein ~2 million years ago (MYA). This expansion or gene conversion coincides with the emergence of the *Homo erectus* (Bar-Yosef et al., 2001).

All human-expressed *TBC1D3* copies carry a human-derived 58 amino acid modification of the carboxy-terminus suggestive of a human-specific neofunctionalization (Guitart et al., 2024). Additionally, the gene family continues to dynamically mutate within the human population. Genome-wide studies have shown that *TBC1D3* is among the most copy number polymorphic and heterozygous gene families in the human species, ranging from 3–39 copies per haplotype genome (Sudmant et al., 2015; Guitart et al., 2024). Using long-read transcriptome sequencing to assign isoforms to specific paralogs, we showed that despite this variability >90% of *TBC1D3* transcription from human fetal brain and induced pluripotent stem cells (iPSCs) maps to Cluster 2, and, more specifically, that over 80% of those transcripts stem from the last *TBC1D3* copy mapping distally at the edge of Cluster 2. We hypothesized that a position effect was responsible for regulating and restricting expression to the edge of Cluster 2.

To test this hypothesis and understand the nature of this potential position effect, we performed a detailed comparative transcriptomic and epigenetic analysis to investigate the regulation of *TBC1D3* in humans and great apes. We find that the Cluster 2 terminal *TBC1D3* paralog dominates expression as the result of fusion with an upstream duplicated gene, *NPEPPSP1*, including its brain-enriched promoter. This *NPEPPS1-TBC1D3* fusion transcript accounts for the majority of *TBC1D3* expression across various tissues. We show that the fusion is mediated by segmental duplication of *NPEPPS* in the common ancestor of African great apes but, by two independent events, was lost in the chimpanzee, bonobo, and gorilla lineages by subsequent gene conversion and deletion events, respectively. Additionally, while both *NPEPPSP1* and *TBC1D3* are copy number polymorphic in humans, we find that the promoter of the fusion gene is fixed in all human genomes examined. Our findings dissect the evolution of the *TBC1D3* position effect and explain how it has become specific to the human lineage providing a model for how new genes rapidly emerge in a species.

## RESULTS

### Matched *TBC1D3* copy expression and regulation in CHM13.

**Figure 1.**
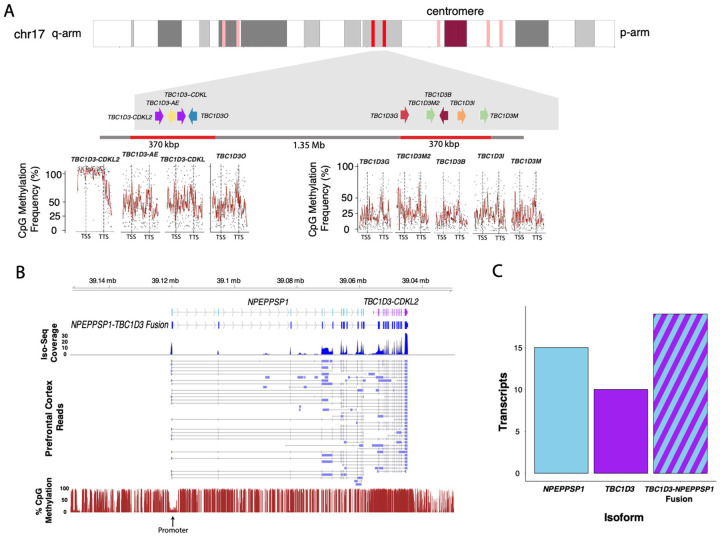
Differential regulation and expression of the human *TBC1D3* gene family. **A.** CpG methylation profile compared among *TBC1D3* paralogs within CHM13 based on mapping of CHM13 ONT data back to the T2T-CHM13 genome (Nurk et al., 2022). Percent methylation of CpG sites from transcription start site (TSS) to transcription termination site (TTS) based on rolling average of 15 consecutive CpG sites (red lines). This methylation signature is consistent with >75% of transcription originating from this distal copy (Guitart et al., 2025). **B.** Expression and promoter definition of the terminal *TBC1D3-CDKL2* copy. A schematic of the *NPEPPSP1-TBC1D3* fusion gene structure, including overall Iso-Seq transcript coverage per exon (top panel) and various isoforms (middle panel) detected based on Iso-Seq transcript data from human prefrontal cortex (Leung et al., 2021). Percent CpG methylation data from CHM13 overlaid with this annotation predicts the likely location of the promoter identified by the dip in methylation (bottom panel). **C.** The absolute number of Iso-Seq transcripts identified as *NPEPPSP1-TBC1D3* fusion vs. solo *TBC1D3* or *NPEPPS1* compared for Iso-Seq data from CHM13.

The high sequence identity (>99%) of *TBC1D3* paralogs has meant that the transcription and regulation of this gene family has essentially been excluded from large-scale studies such as ENCODE (ENCODE Project Consortium, 2012). To resolve this, we initially used matched long-read sequencing (LRS) data from the complete human reference genome (T2T-CHM13) to investigate the methylation profile of the nine *TBC1D3* copies present in this haploid source genome (Fig. 1A; Nurk et al., 2022; Methods). This analysis revealed a striking hypermethylation signature across the gene body of the terminal paralog of Cluster2, *TBC1D3-CDKL2*, whereas all other *TBC1D3* paralogs displayed uniformly low methylation signals, comparable to background levels observed for lowly expressed genes, including SD paralogs (Vollger et al., 2022). Thus, the specific hypermethylation signatures of the gene body *TBC1D3-CDKL2* aligns with one of the canonical epigenetic hallmarks of expressed genes.

To identify the putative promoter driving expression of this distal *TBC1D3-CDKL2* copy, we analyzed the flanking 100 kbp region upstream of its transcription start site (TSS) for evidence of transcription initiation. An analysis of short-read ATAC-seq data and annotated ENCODE cis-regulatory elements in the GRCh38 reference suggested a putative open chromatin accessibility region located 66 kbp from the translation initiation site of *TBC1D3* (Supplementary Fig. 1; ENCODE Project Consortium, 2012). To define the full-length gene structure based on empirical data rather than annotation alone, we mapped full-length Iso-Seq transcriptomic data from human prefrontal cortex (Fig. 1B). Remarkably, 43% (19/44) of transcripts did not align to the canonical RefSeq model but instead represented a fusion isoform with an annotated pseudogene, *NPEPPSP1*, located directly upstream (Fig. 1C). Tracing this dominant isoform to its TSS revealed a distinct dip in methylation immediately upstream of the first exon—a clear hypomethylated promoter signature, consistent with active transcription initiation (Gershman et al., 2022; Vollger et al., 2022).

### Fusion expression and regulation in *in vitro* developing brain model.

**Figure 2.**
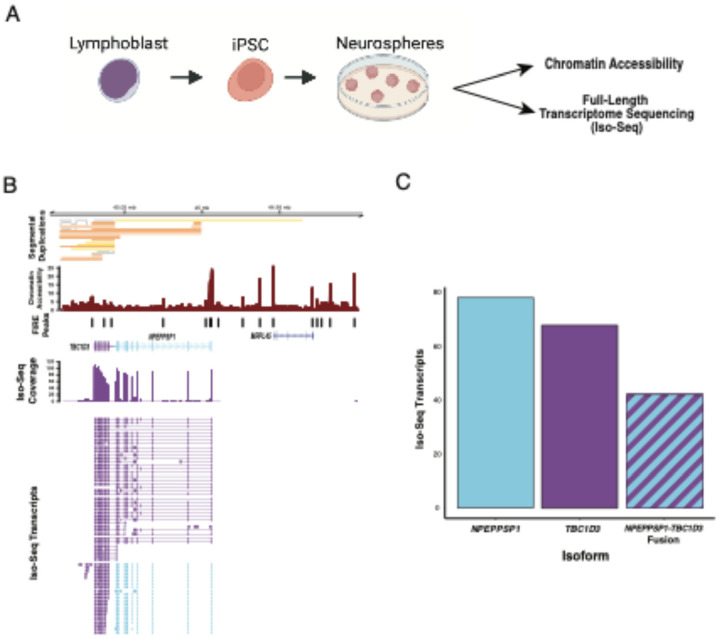
Transcription and chromatin accessibility in human neurospheres. **A.** Schematic depicting neurosphere generation. Lymphoblasts from the 1000 Genomes Project (1KGP) sample, HG02630, were induced to create iPSCs and subsequent neurospheres. Matched Iso-Seq and Fiber-seq data were generated from neurospheres to assess the transcriptome and chromatin accessibility. **B.** Expression and chromatin accessibility of *NPEPPSP1-TBC1D3*. Full-length transcripts mapped to the HG02630 diploid *de novo* sequence assembly are shown with alignment coverage, Fiber-seq accessibility, and segmental duplication sequence annotated with SEDEF (Numanagic et al., 2018). **C.** Absolute expression of *NPEPPSP1*, *TBC1D3*, and the *NPEPPSP1-TBC1D3* fusion. Transcript reads are classified into *NPEPPSP1*, *TBC1D3*, and *NPEPPSP1-TBC1D3* fusion categories.

While CHM13 proved useful to discover the potential site of transcription initiation, this developmental abnormality—a hydatidiform mole—is a poor proxy for the human developing brain, the principal tissue of *TBC1D3* function (Ju et al., 2016; Hou et al., 2021). To investigate *TBC1D3* regulation in a context more relevant to brain development, we first interrogated human neurospheres, an *in vitro* model for neural differentiation harboring both neural progenitor cells (NPCs) and neural stem cells (Fig. 2A; Real et al., 2025). We profiled both chromatin accessibility and transcriptome activity of the *TBC1D3* gene family in neurospheres derived from the HPRC sample HG02630.

Consistent with CHM13 methylation data, this analysis revealed that none of the *TBC1D3* paralogs showed evidence of a proximal promoter—defined as a peak of accessibility directly upstream of the TSS of the canonical gene model (Supplementary Table 1; Stergachis et al., 2020). Instead, the matched transcriptomic-DNA genome assembly data derived from the same neurosphere source material confirmed the initial observation from the analysis of the prefrontal cortex library; 56% (116/206) of primary *TBC1D3* transcript mappings were transcribed as a fusion product with *NPEPPSP1*, over three times the expression than any other individual *TBC1D3* paralog. Using Fiber-seq Inferred Regulatory Element (FIRE) with FIREtools (Fig. 2B; Vollger et al., 2025), we defined the promoter element at the *NPEPPSP1* TSS, a 302 bp unit that regulates expression of the *TBC1D3* paralog located directly downstream of *NPEPPSP1*. Combined, these data suggest that cooption of the *NPEPPSP1* promoter plays a significant role in generating neuronal transcripts of *TBC1D3*, especially from the most distal copy of the *TBC1D3* Cluster 2 (Fig. 2B,C).

### Juxtaposition of novel regulatory DNA by segmental duplication of NPEPPS.

**Figure 3.**
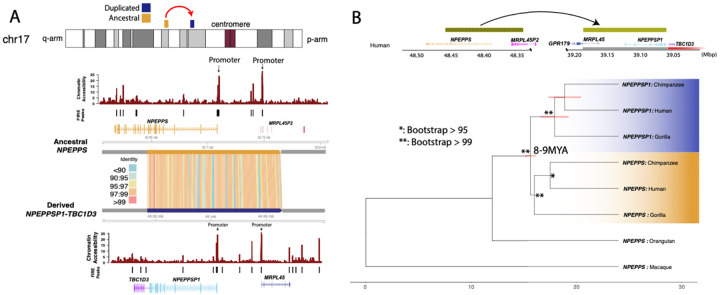
Evolutionary origin of *NPEPPSP1* regulatory sequence. **A.** Comparison of the sequence and regulatory landscape of *NPEPPS* and *NPEPPSP1*. SVbyEye reveals a ~119 kbp SD with 98.2% sequence identity between the ancestral, *NPEPPS*, and duplicated, *NPEPPSP1*, loci. Fiber-seq data analyzed using FIRE compared chromatin accessibility for *NPEPPS* (above) to *NPEPPS1-TBC1D3* (below) with the most likely site of the promoter (indicated). **B.** *NPEPPS1* segmental duplication evolution. A phylogeny of 15 kbp of the *NPEPPS* duplication most proximal to *TBC1D3* and macaque (25 MYA divergence) predicts that the duplication occurred ~8–9 MYA.

To understand the evolutionary origin of the regulatory sequence driving *NPEPPSP1-TBC1D3* expression, we examined its genomic context in the HG02630 diploid assembly. Using SD annotations by SEDEF (Numanagic et al., 2018), the *NPEPPS1* duplication and its associated promoter arose as a 119,358 bp SD originating from *NPEPPS* located 9.16 Mbp distally on chromosome 17q21 (Fig. 3).

A comparison of these two paralogous regions along with corresponding gene annotation of the ancestral locus shows that the promoter sequence of *NPEPPS* (N-peptide puromycin sensitive) matches (Fig. 3A) the promoter sequence associated with *NPEPPS1-TBC1D3* fusion transcripts defined by LRS (Fig. 2). The ancestral *NPEPP*S encodes a zinc metallopeptidase broadly expressed across tissues, and mutations of it have been associated with neurodegenerative tauopathies and Parkinsonism (Henderson Front Gent 2021; Karsten et al., 2006). Thus, the two paralogous promoter pairs regulate expression of *NPEPPS* and *NPEPPSP1*, and *MRPL45* and *MRPL5P1*, respectively (Fig. 3A). The duplicated region shows an average of 98.2% sequence identity by matches only, suggesting a recent origin during ape evolution.

In order to more accurately estimate the timing of the duplication, we extracted a 15 kbp region from the *NPEPPSP1-*anchored end of the SD, directly adjacent to *TBC1D3*. We used this sequence to identify homologous sequences in human, chimpanzee, gorilla, orangutan, and macaque genome assemblies (Yoo et al., 2025). Our analysis shows that the 119 kbp *NPEPPS* SD is duplicated in all African apes assessed (human, chimpanzee, bonobo, and gorilla) (Fig. 3; Supplementary Figs. 2, 3) but is present only as a single unique locus in orangutan and macaque, corresponding to *NPEPPS*. This confirms *NPEPPS* as the ancestral locus and that the duplication (*NPEPPSP1*) arose in the common ancestor of all African great apes. We also constructed a maximum likelihood phylogenetic tree by generating a multiple sequence alignment of all copies among humans and apes using macaque as an outgroup (Fig. 3B). Our analysis estimates, with 100% bootstrap support, that the duplication occurred in the common ancestor of African great apes approximately 8.49 MYA (95% confidence interval of 8.00–9.42 MYA) (Fig. 3B). A comparative analysis of primate genomes (Supplementary Fig. 4) reveals that the *NPEPPS1-TBC1D3* juxtaposition defines the boundary of a series of large inversions, including potential inversion toggling events that break the synteny between the African great apes and other nonhuman primate species.

### Comparative transcriptomics in differentiating neurons.

**Figure 4.**
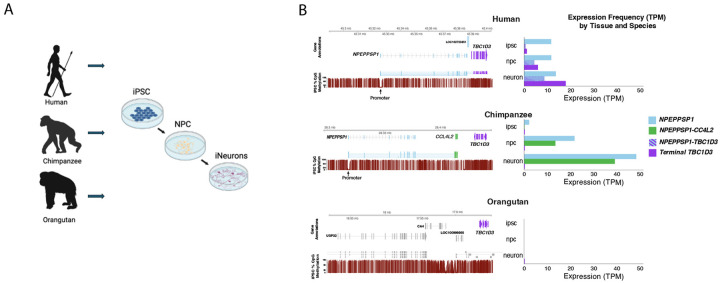
Comparative expression of *NPEPPSP1-TBC1D3* in a neuronal developmental cell culture model of great apes. **A.** Experimental design. Fibroblasts sourced from a human, chimpanzee, and gorilla samples were induced into iPSCs, NPCs, and neurons. Donor-specific genome assemblies from the iPSCs as well as gDNA and mRNA were generated using long-read sequencing protocols for each of the three stages. **B.** Comparative methylation and transcription of *NPEPPSP1*, terminal *TBC1D3*, and fusion genes in human, chimpanzee, and orangutan. A schematic of the gene organization and methylation profile (red; % of read methylated over CpG) is shown for iPSCs from each of the three species (left panels). Expression levels (transcripts per million, TPM) are compared for NPEPPS1, fusion genes, and the terminal TBC1D3 copy for each of three stages (right panels). Histogram illustrates TPM based on Kinnex sequencing (Methods).

To investigate how *NPEPPSP1-TBC1D3* fusion expression changes across neuronal development, and to compare this regulation between humans and other apes, we designed a three-stage cell culture experiment using fibroblast-derived iPSCs from human, chimpanzee, and orangutan (the latter serving as an outgroup; Fig. 4A). Cells were induced sequentially from iPSCs into NPCs, and finally mature neurons with the identity confirmed by marker analyses (Jeong et al., unpublished). At each stage, we collected both genomic DNA (gDNA) and full-length transcriptomes using HiFi sequencing and Kinnex Iso-Seq, respectively. We also generated a donor-specific genome assembly (DSA) for each sample using iPSC gDNA and included methylation calling during base calling to track regulatory changes throughout development. This resource allowed us to match transcript, methylation, and genomic data to specific paralogs without reference bias or cross-mapping among high-identity paralogs.

In humans, we observe stable *NPEPPSP1* expression across all three proxy developmental stages, accompanied by a stepwise increase in *TBC1D3* expression from iPSCs to mature neurons (Fig. 4B). This increase of *TBC1D3* expression was matched by a correlated increase in the *NPEPPSP1-TBC1D3* fusion transcript levels, increasing from a single identifiable fusion transcript in iPSCs to 72% (18/25) and 47% (98/208) of *TBC1D3* expression in NPCs and neurons, respectively. As expected, the fusion transcript was driven by the predicted juxtaposed promoter, characterized by the methylation dip at the *NPEPPSP1* TSS, consistent with the FIRE-defined regulatory elements observed in HG02630 (Fig. 3A; Vollger et al., 2025).

In contrast, chimpanzee *TBC1D3* expression is minimal throughout our model of neuronal differentiation. While both lineages show activation as well as a stepwise increase of the same orthologous *NPEPPSP1* promoter as cells differentiate to neurons, in chimpanzees that stepwise increase in *NPEPPSP1* expression across development is not associated with *TBC1D3* but rather a different gene, *CCL4L2* (CC chemokine ligand 4), a protein associated with inflammation and immune response (Li et al., 2023; Xu et al., 2024). Two key observations help explain the absence of *NPEPPS1-TBC1D3* expression in chimpanzee. First, the downstream *TBC1D3* paralog in the chimpanzee haplotype is inverted with respect to its ORF relative to the *NPEPPSP1* promoter, precluding *NPEPPSP1-TBC1D3* gene expression. Second, we identified a 44 kbp insertion of *CCL4L2* situated between the *NPEPPS1* promoter and *TBC1D3*, resulting in abundant *NPEPPSP1–CCL4L2* fusion transcription in chimpanzees (Fig. 4B). Thus, in chimpanzee, *CCL4L2* appears to co-opt the *NPEPPSP1* promoter analogous to human *TBC1D3* but with a very different outcome. Analysis of the bonobo (*Pan paniscus*) genome indicates that both the inversion and *CCL4L2* insertion are present in this species, suggesting that this organization is a longstanding property of the *Pan* genus likely originating in the common ancestor of the two extant lineages (Supplementary Figs. 2, 3).

Orangutan, which does not harbor the NPEPPS1 SD or its promoter, serves as a negative control and shows scant *TBC1D3* expression of the most terminal copy (Fig. 4B). We note, however, that there is abundant *TBC1D3* expression in the orangutan (Supplementary Fig. 5) from other copies. However, unlike the human ortholog, orangutan *TBC1D3* is uniformly expressed by numerous internal Cluster 2 paralogs that have independently expanded in the *Pongo* genus (Guitart et al., 2024; Supplementary Fig. 6).

**Figure 5.**
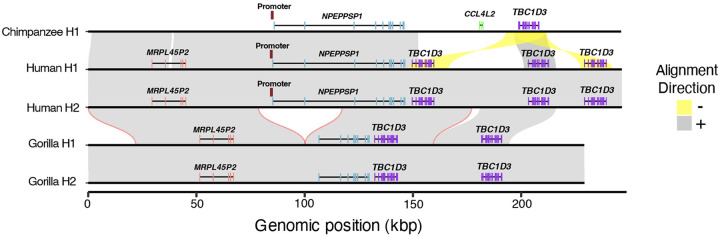
Deletion of gorilla *NPEPPSP1* promoter. Alignments of the *NPEPPSP1* duplication locus between one chimpanzee, two human, and two gorilla haplotypes show a 38 kbp deletion fixed in the gorilla lineage, removing both the *NPEPPSP1* promoter and first two exons.

While gorilla was not included in our neuron development cell model, we also attempted to investigate *NPEPPSP1-TBC1D3* regulation and expression by exploring the orthologous locus in the gorilla genome assembly and available Iso-Seq data from fibroblasts and testis tissue (Yoo et al., 2025). While gorilla inherited the same *NPEPPSP1* duplication, the promoter and first two exons of the pseudogene have been ablated by a subsequent 38 kbp deletion in the gorilla lineage (Fig. 5, Supplementary Fig. 3). Concomitantly, we find no evidence of *NPEPPSP1* promoter-initiated *NPEPPS1-TBC1D3* fusion transcripts. We do find, however, fusion transcripts with transcript initiation starting near the *MRPL45* duplicated promoter, a gene inverted with respect to *TBC1D3* (Supplementary Fig. 7). We observe that these transcripts begin just upstream of the promoter of *MRPL45*, a gene inverted with respect to *TBC1D3*. This regulatory sequence may function as a bidirectional promoter (Trinklein et al., 2004), but *MRPL45* expression is nearly 20-fold higher, with 57 unique *MRPL45* transcripts to the three *TBC1D3* reads.

### The fusion transcript encodes two separate ORFs.

Based on the human *NPEPPSP1-TBC1D3* fusion transcript consensus sequence, we predict two large, mutually exclusive ORFs (Supplementary Fig. 8a). The first ORF (ORF1) comprises a 449-codon reading frame corresponding to the entirety of *NPEPPSP1* and four codons located at the beginning of *TBC1D3* exon 2, traversing the fusion junction. The second ORF begins 13 bases upstream of ORF1, also at the second exon of *TBC1D3*, and represents the canonical 546-aa TBC1D3 ORF annotated by RefSeq (O’Leary et al., 2015). These ORFs are offset by two bases, placing them in different frames, precluding the formation of a fused protein product.

Unlike *TBC1D3*, there has been no characterization of *NPEPPSP1*, a supposed pseudogene, at the protein level. We searched publicly available proteomic resources using an *in silico* tryptic digest of *NPEPPSP1-TBC1D3* ORF1 and found four uniquely identifying peptides distinguishing *NPEPPSP1* from any other protein, including its ancestral paralog, *NPEPPS*. We searched for these four peptides in publicly available mass spectra datasets and identified five proteomic experiments with matches to the peptides. Although not conclusive, these data suggest *NPEPPSP1* may be translated and present in the human proteome (Supplementary Table 2; Methods).

Next, we assessed mutational tolerance of both ORFs by analyzing 295 human genomes recently sequenced as part of the Human Pangenome Reference Consortium (HPRC) (Liao et al., 2023) and the Human Genome Structural Variation Consortium (HGSVC) (Logsdon et al., 2025). Across these haplotypes, we found three instances of a premature termination of *NPEPPSP1*, driven by one splice junction in the third exon, and two nonsense mutations on the seventh exon (Supplementary Fig. 8b). By contrast, we found a single occurrence of a splice junction mutation (3’ ss of exon 10) introducing a premature stop codon in the *TBC1D3* ORF (Supplementary Fig. 8c).

### Posttranscriptional processing of fusion transcripts.

**Figure 6.**
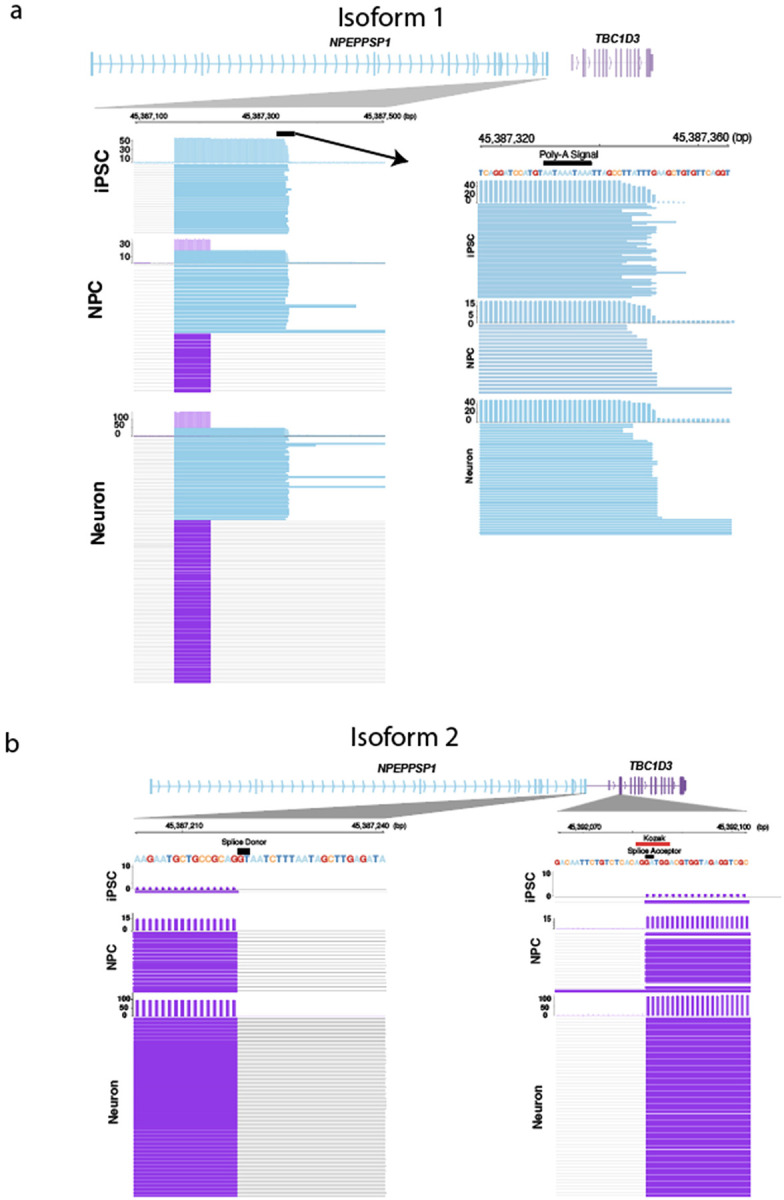
Alternative splicing of *NPEPPSP1-TBC1D3* fusion. **A.** Solo *NPEPPSP1* isoform. Solo *NPEPPSP1* transcripts display a polyadenylation signal (chr17:46387329–46387338) terminating the transcript, a signal that is removed by splicing in fusion genes. **B.** Fusion isoform. *NPEPPSP1-TBC1D3* transcripts display splicing between *NPEPPSP1* exon 10 and *TBC1D3* exon 2, where ORF2 begins. The splice acceptor site of *TBC1D3* overlaps with a Kozak sequence, recognized as AXXATGG.

Because *NPEPPSP1* duplicates only the first 11 exons of *NPEPPS*, we were interested in understanding how this partial duplication impacts the transcription and splicing of the gene. Using our neuronal developmental dataset, we characterized the expression of the two most common isoforms. We observe that solo *NPEPPSP1* transcripts utilize a novel polyadenylation site located 111 bp downstream of the canonical splice junction used by *NPEPPS* (Fig. 6A). This alternative site is strongly favored in iPSCs, being used in >98% (51/52) of full-length transcripts sequenced. The equivalent paralogous poly-adenylation site, however, was not observed in the ancestral *NPEPPS* gene and is excised within the 11th intron by a splice junction upstream of the site. Of note, we find that as cells differentiate toward neurons, *NPEPPSP1* splice site usage shifts toward the equivalent splice junction used by *NPEPPS*, losing the premature polyadenylation site and extending transcription to include the full-length *TBC1D3* ORF (Fig. 6B). Our results suggest that the fusion is regulated posttranscriptionally by alternative splicing of the last and first exons of *NPEPPSP1* and *TBC1D3*, respectively (Fig. 6).

We next sought to understand how the fusion impacts transcript processing. Given that nonsense-mediated decay (NMD) is often triggered by splice junctions located downstream of a translation stop codon, we hypothesized that *TBC1D3-*containing transcripts might be targeted for degradation and, thus, preferentially rescued upon NMD inhibition. To test this, we treated human and chimpanzee iPSCs and NPCs with cycloheximide (a known NMD inhibitor) or dimethyl sulfoxide (DMSO) as a control (Cheng et al., 2025). While we observe an overall increase in expression of *NPEPPSP1*, *TBC1D3*, and the fusion transcript following cycloheximide treatment, we did not observe preferential rescue of *TBC1D3*. Instead, *NPEPPSP1* transcripts show the most pronounced increase (Supplementary Fig. 9), suggesting that they may be more susceptible to NMD than *NPEPPS1-TBC1D3* fusion transcripts.

Importantly, we find that *TBC1D3* expression surpasses *NPEPPSP1* in transcript abundance as human cells differentiate into neurons (Supplementary Figs. 10–12; Supplementary Tables 3,4). Initially, we considered the possibility that this was due to expression from other *TBC1D3* paralogs. However, both epigenetic profiling and full-length transcript analysis across paralogs rule out this explanation. All 196 transcripts map to the terminal Cluster 2 *TBC1D3* copy. Only a single hypomethylation signature is identified corresponding to the promoter of the fusion transcript. We fail to identify any methylation or chromatin accessibility signatures consistent with a promoter in another paralog (Supplementary Tables 1,5).

While it is possible that this neuronal enrichment of *TBC1D3* may be due to the moderate 3’ bias of the Kinnex and Iso-Seq LRS platforms, this seems unlikely since it is not observed in either NPCs or iPSCs, which were processed identically with respect to capture. We speculate, therefore, that there may be a posttranscriptional mechanism that isolates the downstream *TBC1D3* reading frame from its upstream linked partner, *NPEPPSP1*. In support of this, we identified a Kozak sequence, AXXATGG, in the second exon of *TBC1D3*, located at the acceptor site of the splice junction fusing both genes, and including the start coding of ORF2. This site may serve as an internal ribosomal entry site for translation (Fig. 6B; Hellen, 2001).

## DISCUSSION

This work advances our understanding of *TBC1D3* regulation and evolution in four major ways. First, we demonstrate that *TBC1D3* expression in humans is regulated via a position effect, primarily as a result of a fusion duplication event with the upstream duplicated gene, *NPEPPSP1*. Paralog-specific regulation via position effect is a well-documented phenomenon, as exemplified in the locus control region (LCR) regulating immediately proximal green and red opsin paralogs in chromosome X, and none of the additional copies downstream (Hayashi et al., 1999). Other examples include the beta-globin LCR (Reik et al., 1998) and the 5’ LCR in *hGh* (Ho et al., 2006). Analogously, *NPEPPS1* defines an LCR of the terminal paralog of *TBC1D3* Cluster 2 with other paralogs being epigenetically repressed (Figs. 1 and 2).

Second, we identify the origin of this regulatory fusion as an SD event that duplicated ~120 kbp—encompassing the *NPEPPS* promoter and first 11 exons. The ancestral *NPEPPS* locus maps 9.16 Mbp proximally to the *TBC1D3* Cluster 2 locus on chromosome 17 (Fig. 3). Gene fusion arising from the juxtaposition of partial SDs has been proposed as a relatively common mechanism of neofunctionalization, particularly in primates (Marques-Bonet, Girirajan et al., 2009; Marques-Bonet, Kidd, et al., 2009; Yoo et al., 2025). For example, *HYDIN2* arose from partial duplication of *HYDIN*, resulting in a novel gene fused to *LOC101927468* (Dougherty et al., 2017) and *CHRFAM7A* (i.e., *dupα7*), a human-specific fusion *CHRNA7* exons 5–10 and *FAM7A* exons A-E, regulating *CHR7A* in a dominant negative fashion (Sinkus et al., 2016).

In humans, the terminal copy of *TBC1D3* in Cluster 2 co-opted the regulatory program of *NPEPPS* to drive transcription of a fusion transcript. *NPEPPS*, also known as puromycin-sensitive aminopeptidase (PSA), is a cytoplasmic aminopeptidase conserved across metazoans that is broadly expressed across tissues and plays a key role in protein metabolism and cell cycle regulation (McLellan et al., 1988). In the brain, NPEPPS is enriched in the cerebellum and hippocampus, where it provides a protective effect against tauopathies and associated neurodegenerative diseases, including Parkinson’s and dementia (Karsten et al., 2006; Muraoka et al., 2020). Disease associations remain difficult for *TBC1D3*, owing to the high sequence identity between paralogs and the significant degree of structural heterozygosity among individuals (Guitart et al., 2024). However, we find that *NPEPPSP1-TBC1D3* expression is enriched in the brain, and in particular, increases in expression in the cerebellum as the brain develops into adulthood (Supplementary Fig. 13). *In vitro* experiments of *TBC1D3* propose numerous mechanisms of *TBC1D3* function, all of which increase cell proliferation (Frittoli et al., 2008; Wainszelbaum et al., 2008, 2012; Ju et al., 2016; Hou et al., 2021). The human cerebellum is unique among other brain regions for its delayed maturation and high degree of neuronal density, which holds ~80% of brain neurons despite constituting only 10% of volume (Van Essen et al., 2018). Recent *in vitro* studies suggest *TBC1D3* both promotes neuronal proliferation and protracts synaptic plasticity and contributes to neoteny (Hou et al., 2021; Dong et al., 2024). We suggest *TBC1D3* may contribute to human cerebellar development through this novel transcriptional context or the human-specific carboxy-terminus (Guitart et al., 2024).

Gene fusions driven by transcriptional readthrough commonly coincide with the fusion of two reading frames into a single novel protein (McCartney et al., 2019). In contrast, *NPEPPSP1-TBC1D3* retains two large, independent reading frames across the singly transcribed fusion (Supplementary Fig. 8). Polycistronic expression has been documented in eukaryotes, restricted, however, almost exclusively to mitochondrial and plastid genes—both derivatives of prokaryotes (Barkan et al., 1988; Mercer et al., 2012). However, multi-ORF genes have been characterized. *ATF4* and *GRN*, for example, include an upstream ORF (uORF) whose retention in the transcript either promotes or inhibits translation of the downstream, main ORF (mORF) (García-Ríos et al., 1997; Ryczek et al., 2023; Vattem et al., 2004; Capell et al., 2013). In these cases, however, the uORFs tend to be small, only 16 aa on average (Wethmar et al., 2010), while the reading frame we observe for NPEPPSP1 is 479 aa.

Third, we illustrate a developmentally dependent, posttranscriptional regulation of *TBC1D3* as cells differentiate into neurons. In our neuronal differentiation system, *TBC1D3* increases in expression and fusion transcripts proportionally diminish as human stem cells differentiate into neurons (Fig. 6; Supplementary Figs. 10–12), raising the possibility of independent expression of *TBC1D3*, possibly from other paralogs, or the removal of *NPEPPSP1* from the fusion gene via alternative splicing. Our epigenetic profiling reveals that nonterminal paralogs remain epigenetically silenced, lacking the promoter-associated hypomethylation and chromatin accessibility (Figs. 1 and 2). We therefore propose that the *TBC1D3* reading frame is selectively retained in the transcript via a posttranscriptional process that removes or excludes the upstream *NPEPPSP1* ORF. Supporting this, cycloheximide treatment, which inhibits NMD, selectively rescues *NPEPPSP1* and fusion transcripts when compared to *TBC1D3* (Supplementary Fig. 9). This suggests a posttranscriptional regulatory model in which neuronal cell fates isolate *TBC1D3*, potentially influencing its translation efficiency or subcellular localization. Follow-up protein-level studies (e.g., immunofluorescence or mass spectrometry) are needed to assess the fate of the protein(s) derived from the NPEPPSP1-TBC1D3 fusion transcript.

Finally, we place this regulatory architecture in an ape evolutionary framework. The *NPEPPSP1* duplication event occurred between 8–9 MYA, in the common ancestor of African apes. This time interval corresponds to a period of rapid chromosomal restructuring, including large-scale inversions and fusions that dramatically restructured local chromosomal regions in different ape lineages potentially as a result of extensive incomplete lineage sorting (Yang et al., 2025; Mao et al., 2021, Yoo et al., 2025). The *NPEPPSP1-TBC1D3* locus exhibits structural divergence across apes, with incomplete lineage sorting and lineage-specific rearrangements contributing to distinct haplotypes in humans, chimpanzees, and gorillas, including multiple large-scale and smaller inversion events (Supplementary Figs. 2,3). Despite the shared origin of the fusion in all African great-ape lineages, *TBC1D3* expression driven by *NPEPPSP1* appears to be human-specific. In the genus *Pan*, a subsequent 44 kbp chimpanzee duplication inserted *CCL4L2* between *NPEPPSP1* and *TBC1D3*, disrupting the fusion in both chimpanzees and bonobos. In gorilla, a 51 kbp deletion removed the *NPEPPSP1* promoter and initial exons, ablating the fusion transcript. Based on the genomic architecture, only humans are, thus, capable of producing the *NPEPPSP1-TBC1D3* fusion product at high levels during neurodevelopment.

We find that the expressed *TBC1D3* gene has been dramatically restructured during human evolution. Our previous work identified a human-specific C-terminal truncation resulting in a 58-aa modification in all expressed human copies. Here, we show that the 5’ end of the major human *TBC1D3* transcript is also unique to our lineage—it includes the first 11 exons of *NPEPPSP1*. Further, we provide evidence that this modification boosts expression during neuronal development. The functional consequences of these alterations—for translation, localization, or function—remain unresolved. Experimental perturbation of *TBC1D3* remains challenging due to its high sequence identity across paralogs. However, our findings present a potential solution: targeting the fixed *NPEPPSP1* promoter, which regulates the polymorphic *TBC1D3* cluster through a position effect. This regulatory mechanism—where a fixed regulatory element controls expression of structurally variable loci—may be more common than expected, offering a new opportunity to understand, and eventually target, structurally variable and medically relevant genes that have, until recently, been impossible to characterize.

## METHODS

### Methylation Analysis

ONT BAM data used for the CHM13 methylation analysis were generated and processed described in (Mastrorosa et al., 2024). CHM13 genomic DNA reads used to generate the T2T-CHM13 reference were aligned back onto the reference using minimap2 (version 2.28; Li et al., 2018). Following alignment, methylation base calls of CpG sites were processed and aggregated using the modbam2bed software (v0.10.0; github.com/epi2me-labs/modbam2bed). Following methylation aggregation, CpG sites with coverage of five reads or less were discarded. For visualization (see Fig. 1), percent of reads methylated over each CpG site are illustrated as black points, with a rolling average of 15 CpG sites visualized with a red line. These results were visualized in R using ggplot2 geom_point and geom_line functions.

Similarly, methylation calling for ONT fast5 files from iPSCs, NPCs, and iNeurons, generated from human, chimpanzee, and orangutan, respectively, was generated during base calling by Dorado (version 0.8.2). These reads were then aligned to the respective species DSAs, characterized in (Jeong & Yoo, in preparation) with minimap2 (version 2.28). Methylation calls were then aggregated over CpG sites with Modkit (version 0.4.1).

### Fiber-seq Analysis

Fiber-seq libraries of HG02630 were prepared as described in (Real et al., 2025). Reads from the dataset were realigned to polished HG02630 assemblies generated with Deep polisher by the HPRC (Mastoras et al., 2025). Reads were mapped back to HG02630 with the following minimap2 (version 2.24), and adenosine and cytosine methylation calls from the original base calling were maintained with the new alignment BAMs. FIRE peaks were identified using FIREtools (version 0.0.7; Vollger, et al., 2025). Fiber-seq peaks were visualized with Gviz (version 1.46.1).

### Full-Length Transcript Analysis

CDNA libraries were aligned as previously reported in (Guitart et al., 2024). Reads were aligned to a reference assembly using minimap (version 2.24) with the settings <minimap2 -ax splice --sam-hit-only --secondary=yes -p 0.5 --eqx -K 2G>. Following alignment, primary and secondary alignments were scrutinized. For paralog-specific reads, primary alignments with alignments scores greater than second-best alignments were used. Reads were visualized with R using the Gviz visualization library. Reads mapping to only a single exon were excluded from the gene transcript counts.

Iso-Seq libraries from multiple tissue sources were gathered as described in (Dishuck et al., 2025). Iso-Seq libraries were normalized to transcripts per million. Read counts were then organized by hierarchical clustering with R hclust and visualized as a heat plot with ggplot2.

### Polyadenylation Analysis

Polyadenylation signals were identified by looking for a canonical polyadenylation motif (AATAAA) within 20 bp of the end of transcript read alignments (Higgs et al., 1983). Splice junctions were identified by marking AG 3’ splice acceptor and GT 5’ splice acceptor motifs at splice junctions along cDNA alignments (Stephens & Schneider 1992). Polyadenylation signals and splice junctions were visualized with rtracklayer (version 1.62.0; Lawrence et al., 2009).

### Phylogenetic Analysis

Multiple sequence alignments (MSAs) for phylogenies were generated as follows. Homologous sequence was identified by mapping a reference target sequence to assemblies using minimap2 (-x asm20 --secondary --eqx -p −.05 -N 1000). Following sequence extraction, homologous sequences were aligned as an MSA with MAFFT (version 7.5.25; Katoh et al., 2013) with the following parameters: --reorder --preservecase --adjustdirection --maxiterate 1000 --thread 16. Following MSA generation, maximum likelihood phylogenies were generated with iqtree2 (version 2.1.2; Minh et al., 2020) with the following parameters: -nt AUTO -m MFP -s {MSA_fasta} -o {outgroup} --prefix {output_name} -alrt 1000 -bb 1000.

For *NPEPPSP1* duplicon phylogenetic analysis, the terminal 15 kbp of the *NPEPPSP1* duplication most proximal to *TBC1D3* was extracted using SEDEF annotations (Numanagic et al., 2018). The sequence was then mapped to the human, chimpanzee, gorilla, orangutan, and macaque telomere-to-telomere (T2T) assemblies using minimap2 with the default setting “-x asm20” (Yoo et al., 2025). These mappings were extracted with BEDTools and aligned into an MSA with MAFFT (version 7.525; Katoh et al., 2013). Next, the MSA was trimmed for gaps and misalignments with both Trimal (version v1.4.rev22) and manual pruning. Then, a phylogeny and timing estimates were generated using the iqtree2 software (version 2.12; Minh et al., 2020), with macaque as the outgroup. Timing estimates were calculated with iqtree2, using the following divergence times: (human–chimpanzee: 6.5 MYA; human–gorilla: 8.0 MYA; human–macaque: 24 MYA) as estimated in the fossil record (Dunsworth 2010; Stevens et al., 2013). The following command for timing estimation was used: iqtree2 -s {MSA} --date {divergence_date list} --date-ci 100 -te {phylogeny } --keep-ident -redo -o {outgroup_name} --date-tip 0 -alrt 1000 -bb 1000). For *CCL4L2*, the same process described as above was followed for the alignment and characterization of the *CCL4L2* gene model with 5 kbp of upstream sequence included.

### Short-Read-Depth Analysis

Short-read libraries from the 1000 Genomes Project (**Sudmant et al., 2010**). Illumina libraries were K-merized into 32 bp libraries with Meryl (version 1.4.1). Next, k-mer libraries were aligned to the T2T-CHM13 reference genome (Nurk et al., 2022) using FastCN (Pendleton et al., 2018), allowing for up to two mismatches between 32-mer and assembly alignments. Copy numbers over *TBC1D3*, *NPEPPSP1*, and the *NPEPPSP1* inferred promoter (Supplementary Fig. 13) were estimated by taking the average copy number over the given region, estimated by normalized read-depth coverage to unique diploid sequence across the genome.

### Conservation in Human Haplotypes

Gene sequences were aligned using an MSA with MAFFT (v7.525). Single-nucleotide variants (SNVs) and indels were identified by comparing each gene sequence to the reference gene sequence from T2T-CHM13v2.0. Variant functional consequences were annotated using VEP (v.111) with gene annotation (JHU RefSeqv110 + Liftoff v5.2; Shumate et al., 2021). Pathogenicity predictions for missense variants were obtained using AlphaMissense (Cheng et al., Science, 2023), which were lifted over to T2T-CHM13 coordinates using UCSC LiftOver and complemented by PolyPhen and SIFT (version 20240502). Lollipop plots were generated using the R package trackViewer (v1.40.0).

### Haplotype Sequence Annotation

SDs for *NPEPPS-TBC1D3* haplotypes were annotated in the *de novo* assemblies with SEDEF (version 1.1; Numanagic et al., 2018). Duplicon tracks were generated for each assembly with DupMasker (Jiang et al.) and run with the Rhodonite workflow (version 0.12; https://github.com/mrvollger/Rhodonite).

### RNA Analysis

Short-read libraries from (Coorens et al., 2025) were used for the analysis. Reads were aligned to T2T-CHM13 using BWA (version 0.7.17), and transcript abundance was normalized using pydeseq2 (version 0.5.2). Read abundance for *NPEPPSP1-TBC1D3* fusion expression was specifically quantified by counting reads spanning the merged exon boundaries across the last and first exons of *NPEPPSP1* and *TBC1D3*, respectively.

### Reading Frame Maintenance

Gene sequences were aligned using an MSA with MAFFT (v7.525). SNVs and indels were identified by comparing each gene sequence to the reference gene sequence from T2T-CHM13v2.0. Variant functional consequences were annotated using VEP (v111; McLaren et al., 2016) with gene annotation (JHU RefSeqv110 + Liftoff v5.2). Pathogenicity predictions for missense variants were obtained using AlphaMissense (Cheng et al., Science, 2023), which were lifted over to CHM13 coordinates using UCSC LiftOver and complemented by PolyPhen and SIFT (20240502). Lollipop plots were generated using the R package trackViewer (v1.40.0).

### Mass Spectrometry Analysis

Amino acid sequences for *NPEPPSP1* and *NPEPPS* were pulled from RefSeq annotations and aligned into an MSA with MAFFT (v7.525). Amino acid substitutions between the two paralogs were determined visually from the alignment. Next, tryptic peptides for both protein sequences were computationally inferred with the Expasy online tool (https://web.expasy.org/peptide_cutter/). Diverse peptide sequences between the two proteins were then selected and isolated for later analysis. These peptides were searched for in the MassIVE database (https://massive.ucsd.edu/ProteoSAFe/static/massive.jsp?redirect=auth).

## Supplementary Material

1

## Data Availability

CHM13 methylation data was previously described in Nurk et al., 2022. Prefrontal cortex Iso-Seq data mapped to T2T-CHM13 was previously reported in Leung et al., 2021, with data available on SRA under BioProject identification number PRJNA664117. Fiber-seq data was previously reported in Real et al., 2025, and deposited on SRA under BioProject identification number PRJNA1236375. HG02630 assemblies used for Fiber-seq analysis were previously reported in Mastoras et al., 2025. Iso-Seq and Kinnex libraries explored in Supplementary Fig. 13 were previously described in Dishuck et al., 2025. Gorilla testis and fibroblast Iso-Seq data described in Supplementary Fig. 7 were previously reported in Guitart et al., and is available as part of the NCBI BioProject PRJNA902025. Primate assemblies investigated were generated as part of the T2T and primate T2T projects, reported in Nurk et al., 2022, and Yoo et al., 2025. *De novo* assemblies and transcriptomic sequence generated for human, chimpanzee, and orangutan iPSCs, NPCs, and iNeurons, were developed and reported in (Jeong et al., in preparation). Short read gDNA data used for WSSD investigation was previously reported in Sudmant et al., 2010.
