# Supplementary material for: NPEPPS segmental duplication drives position effect expression of TBC1D3 in the human brain
Source: bioRxiv. 2026 Jan 15:2026.01.14.699559. Preprint. [Version 1] doi: 10.64898/2026.01.14.699559 (PMC12871316; doi:10.64898/2026.01.14.699559)
Supplement: 1 [file NIHPP2026.01.14.699559V1-supplement-1.pdf]

bioRxiv preprint doi: https://doi.org/10.64898/2026.01.14.699559; this version posted January 15, 2026. The copyright holder for this preprint (which was not certified by peer review) is the author/funder, who has granted bioRxiv a license to display the preprint in perpetuity. It is made available under aCC-BY 4.0 International license.

## SUPPLEMENTARY FIGURES

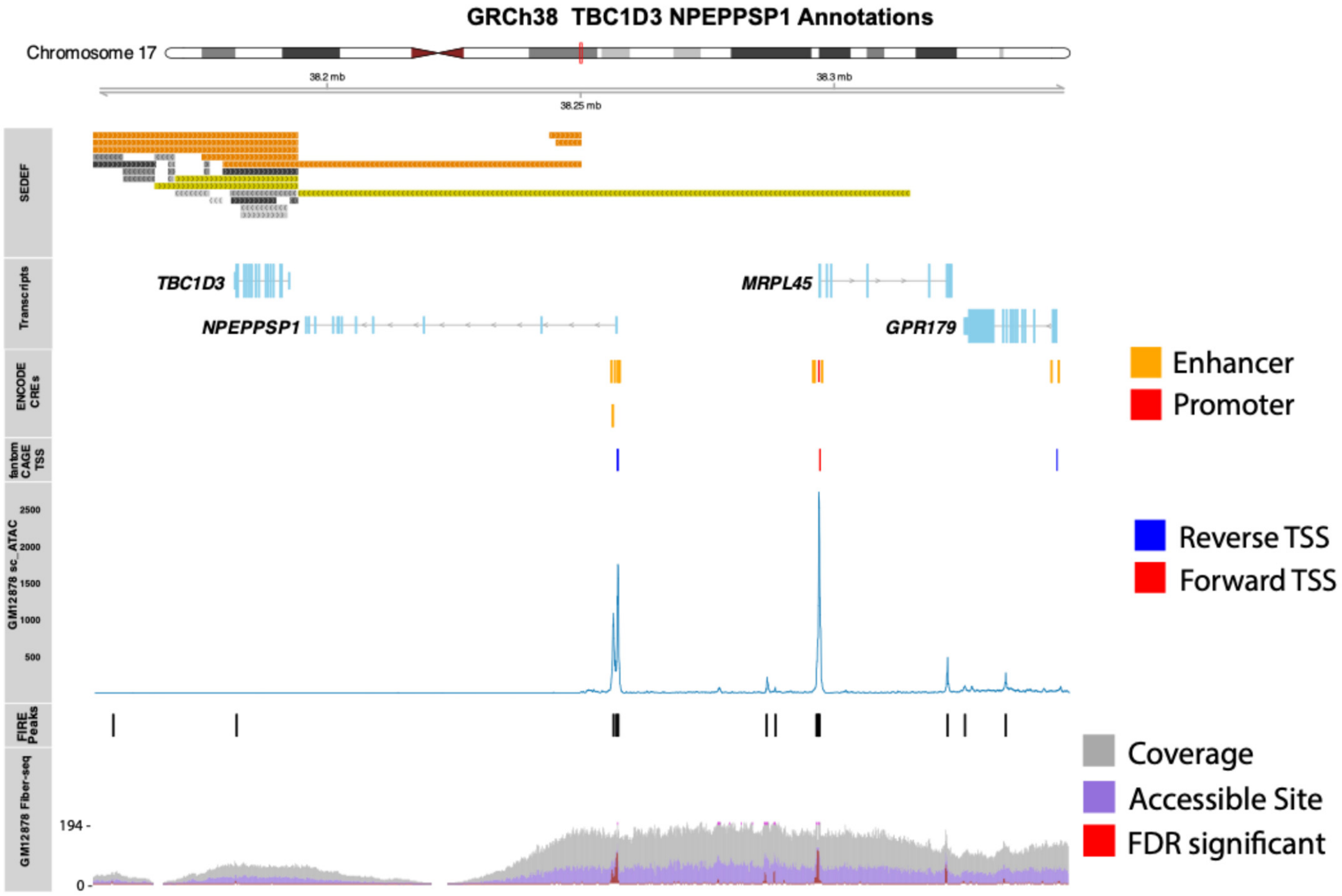


**<u>Supplementary Figure 1. Gene regulatory elements in GRCh38.</u>** Regulatory elements identified by the ENCODE project and scATAC are compared to Fiber-seq data of the GM2878 cell line (Vollger et al., 2025). TSS: transcription start site; CRE: cis-regulatory element.

bioRxiv preprint doi: https://doi.org/10.64898/2026.01.14.699559; this version posted January 15, 2026. The copyright holder for this preprint (which was not certified by peer review) is the author/funder, who has granted bioRxiv a license to display the preprint in perpetuity. It is made available under aCC-BY 4.0 International license.

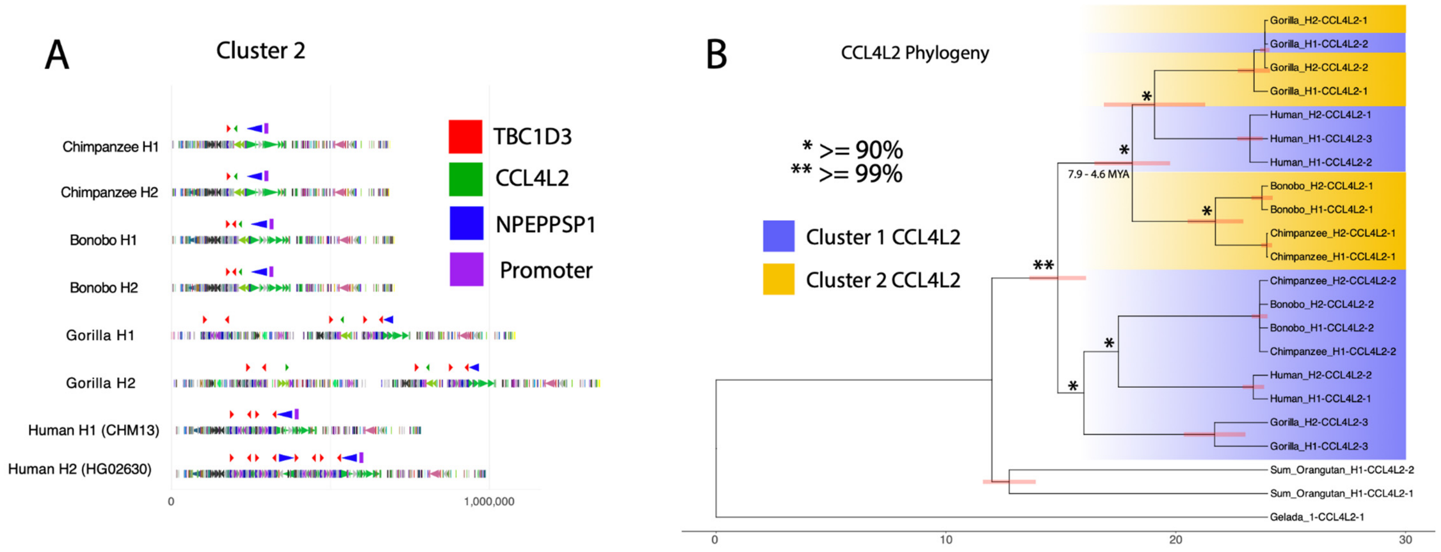


**<u>Supplementary Figure 2. *CCL4L2* structure and phylogeny.</u> A.** Genomic architecture of *TBC1D3* Cluster 2 in African apes. *CCL4L2* obstructs *NPEPPSP1* from *TBC1D3* and co-opts fusion expression in the *Pan* genera. This structure is fixed in both chimpanzee and bonobo. **B.** Maximum likelihood phylogeny of *CCL4L2* sequence. Timing estimates of the *CCl4L2* obstruction event are between 4.6 and 7.9 MYA, and human and gorilla *CCL4L2* copies are more similar to one another than *Pan*.

bioRxiv preprint doi: https://doi.org/10.64898/2026.01.14.699559; this version posted January 15, 2026. The copyright holder for this preprint (which was not certified by peer review) is the author/funder, who has granted bioRxiv a license to display the preprint in perpetuity. It is made available under aCC-BY 4.0 International license.

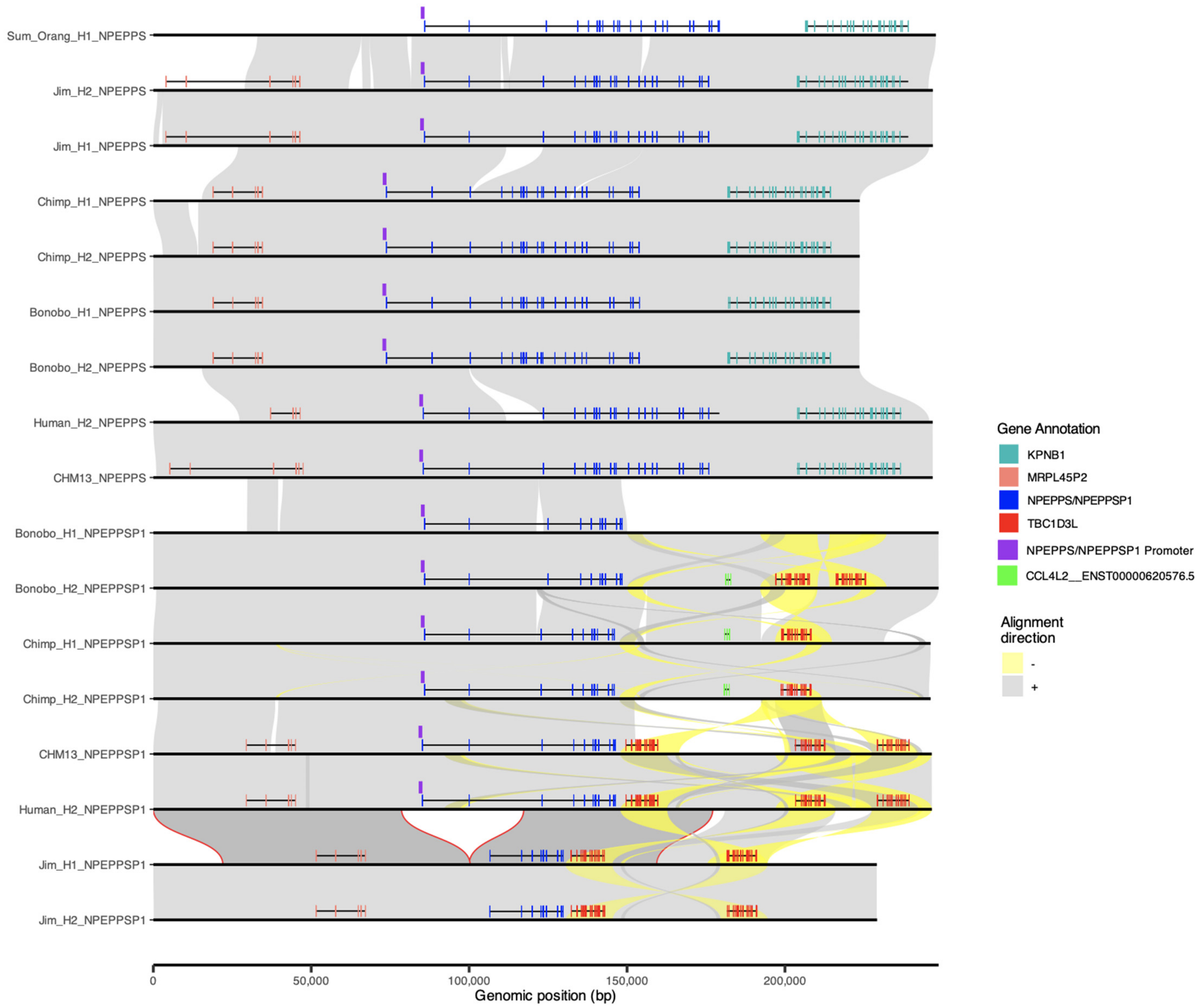


**Supplementary Figure 3. Gorilla deletion.** Alignments of the *NPEPPS* (ancestral) and *NPEPPSP1-TBC1D3* (derived) loci are illustrated, showing the orthologous *NPEPPSP1* promoter inherited across African apes but lost in the gorilla lineage.

bioRxiv preprint doi: https://doi.org/10.64898/2026.01.14.699559; this version posted January 15, 2026. The copyright holder for this preprint (which was not certified by peer review) is the author/funder, who has granted bioRxiv a license to display the preprint in perpetuity. It is made available under aCC-BY 4.0 International license.

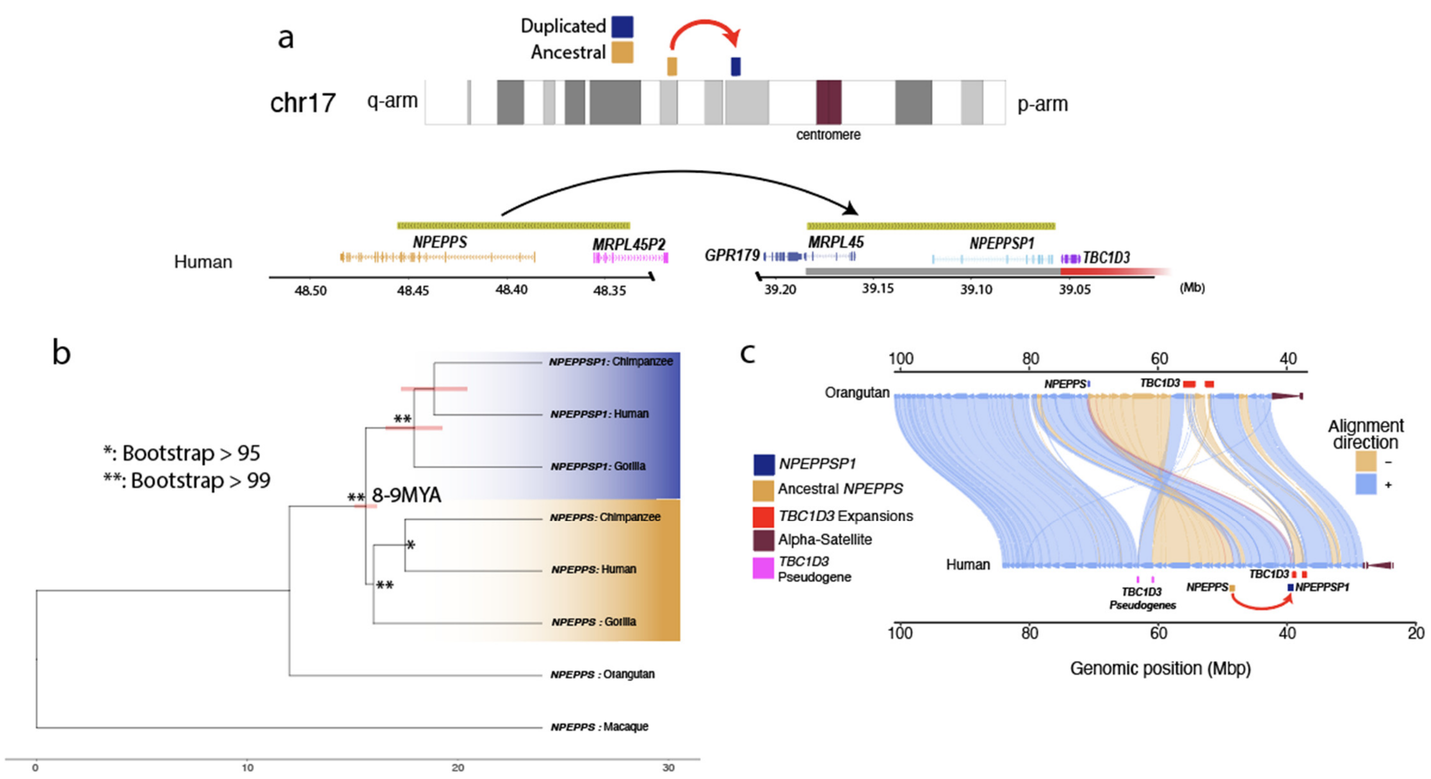


**<u>Supplementary Figure 4. Evolutionary origin of *NPEPPSP1* regulatory sequence.</u>** **A.** *NPEPPS* SD. A ~119 kbp duplication of the N-terminus of *NPEPPS* and C-terminus *MRPL45P1* relocated ~9.15 Mbp to the *TBC1D3* Cluster 2 region. **B.** *NPEPPS1* SD evolution. A phylogeny of 15 kbp of the *NPEPPS* duplication most proximal to *TBC1D3* and macaque (25 MYA divergence) predicts that the duplication occurred ~8-9 MYA. **C.** SVbyEye illustrates the repositioning of *NPEPPSP1* to *TBC1D3*.

bioRxiv preprint doi: https://doi.org/10.64898/2026.01.14.699559; this version posted January 15, 2026. The copyright holder for this preprint (which was not certified by peer review) is the author/funder, who has granted bioRxiv a license to display the preprint in perpetuity. It is made available under aCC-BY 4.0 International license.

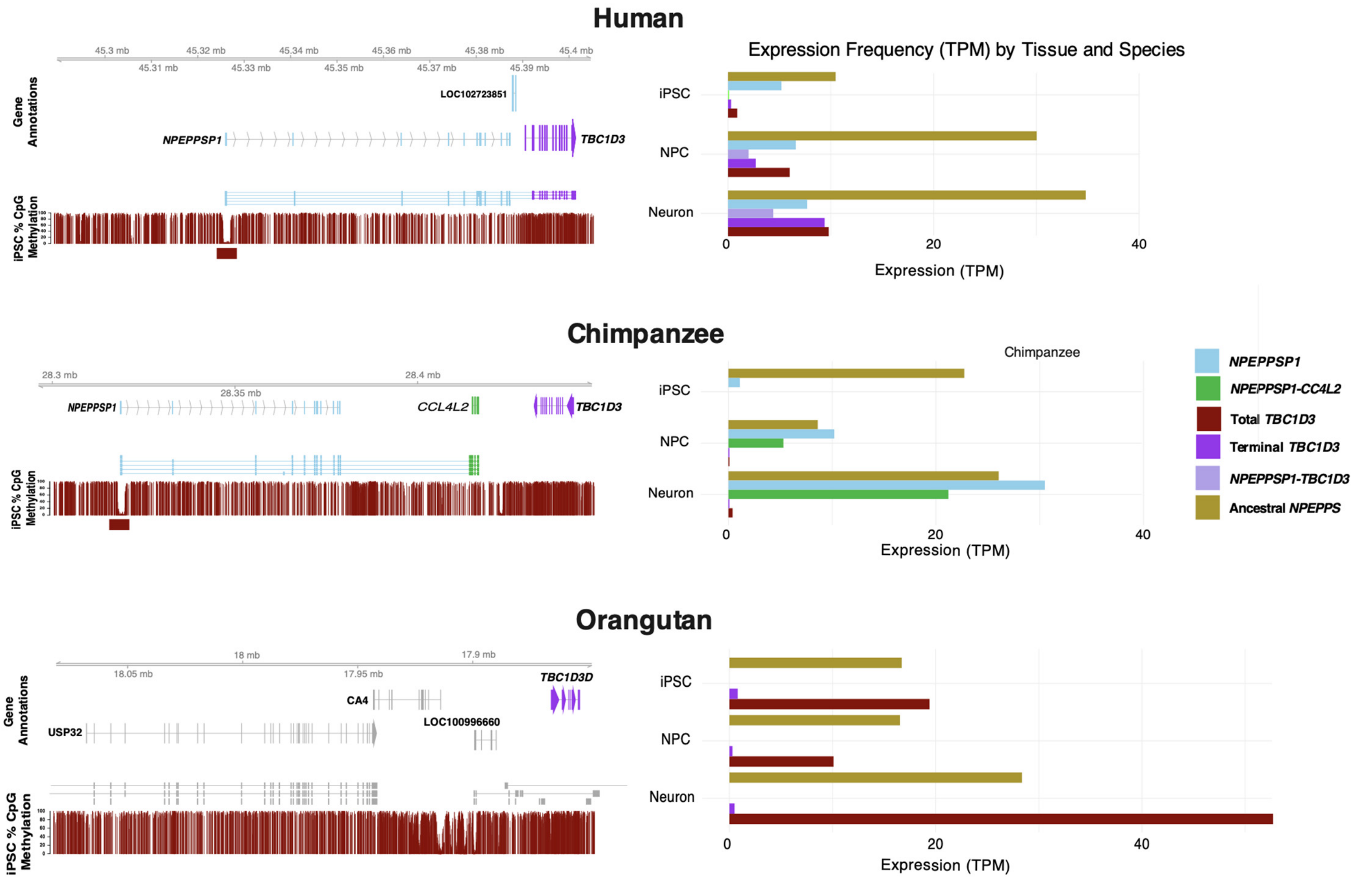


**Supplementary Figure 5. Comparative expression with *NPEPPS* and total *TBC1D3* in a neuronal developmental cell culture model of great apes.** Gene annotation and methylation of *NPEPPSP1-TBC1D3* can be observed on the left for human (top), chimpanzee (middle), and orangutan (bottom). On the right, expression of *NPEPPSP1*, terminal *TBC1D3*, and their fusion are compared to global *TBC1D3* and *NPEPPS* expression, normalized as transcripts per million (TPM; Methods).

bioRxiv preprint doi: https://doi.org/10.64898/2026.01.14.699559; this version posted January 15, 2026. The copyright holder for this preprint (which was not certified by peer review) is the author/funder, who has granted bioRxiv a license to display the preprint in perpetuity. It is made available under aCC-BY 4.0 International license.

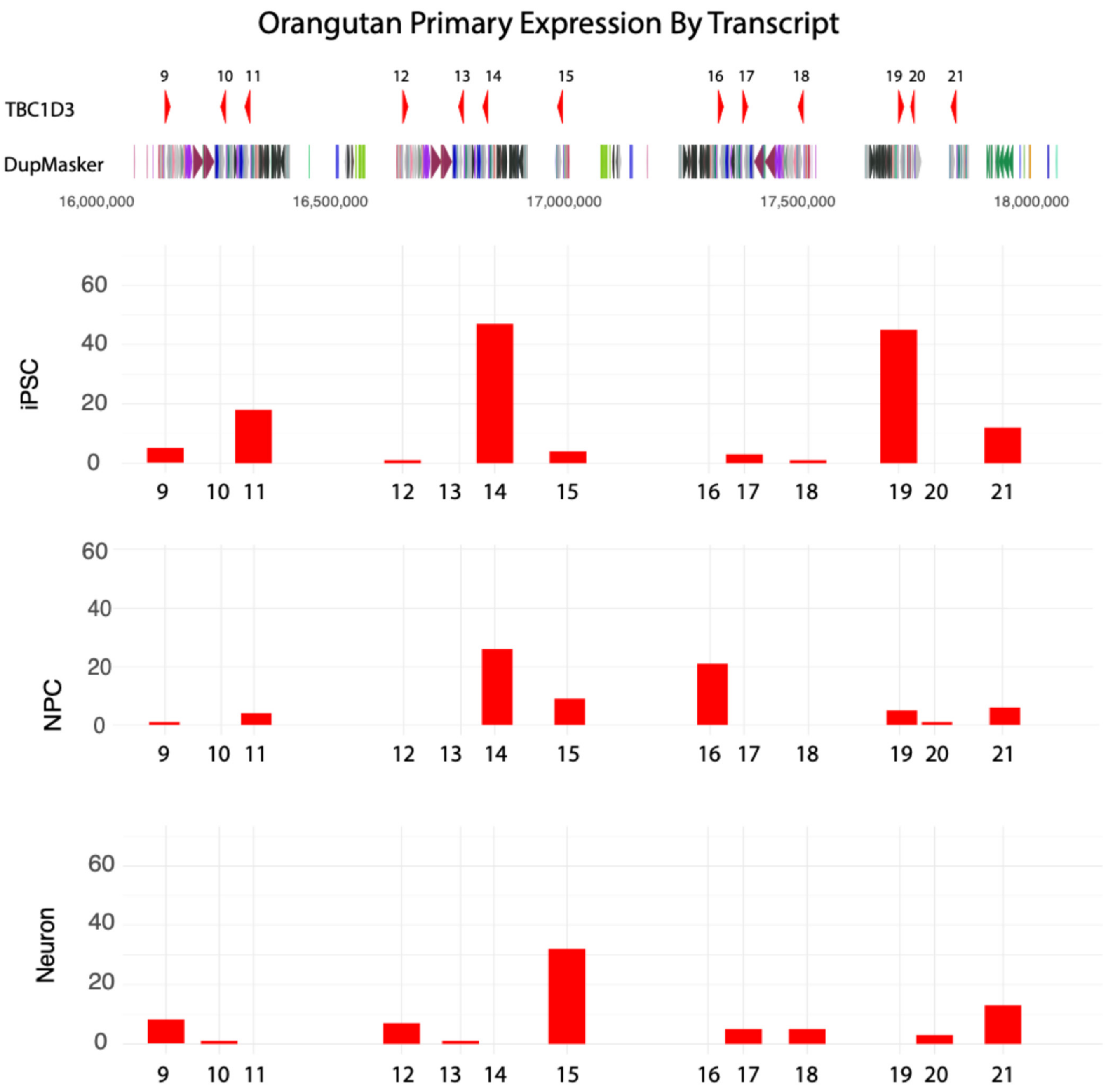


**<u>Supplementary Figure 6. Orangutan expression by *TBC1D3* transcript.</u>** Orangutan primary transcripts map to numerous internal *TBC1D3* paralog copies (*TBC1D3*-14,15,16,19).

bioRxiv preprint doi: https://doi.org/10.64898/2026.01.14.699559; this version posted January 15, 2026. The copyright holder for this preprint (which was not certified by peer review) is the author/funder, who has granted bioRxiv a license to display the preprint in perpetuity. It is made available under aCC-BY 4.0 International license.

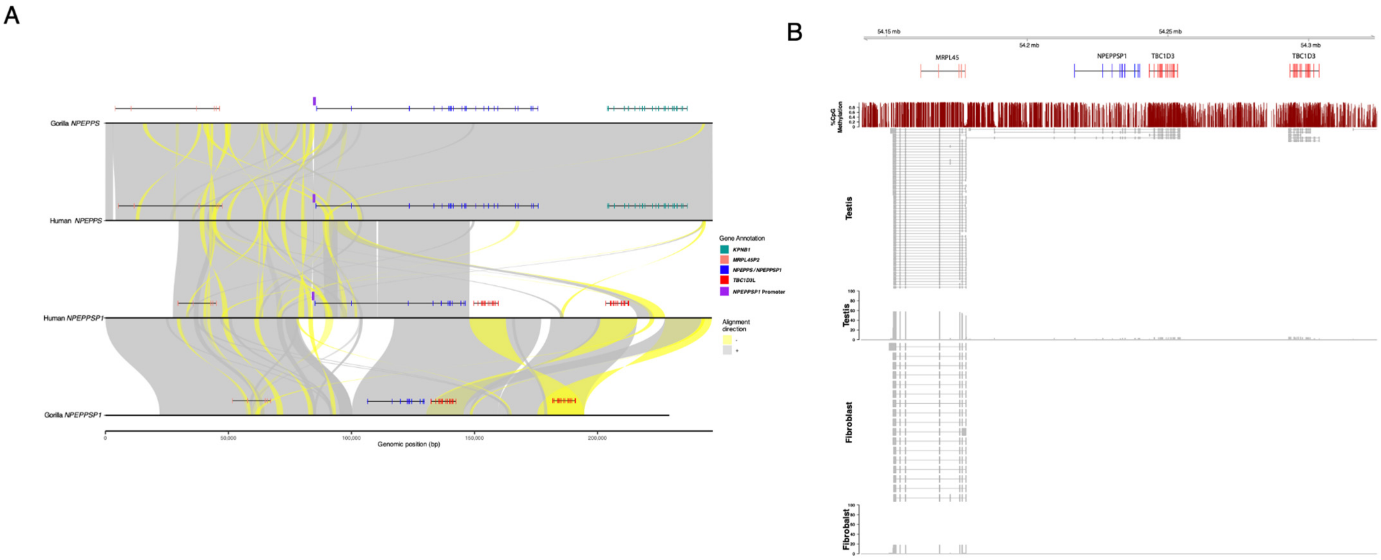


**<u>Supplementary Figure 7. Gorilla *NPEPPSP1* promoter deletion and *TBC1D3* expression.</u> A.** Comparison of gorilla and human genome organization for *NPEPPSP1-TBC1D3* (SVbyEye) highlights a 38 kbp deletion removing the promoter and two exons of *NPEPPSP1* in both haplotypes of the gorilla. **B.** Gorilla isoforms and expression. Mapping of gorilla Iso-Seq data to the gorilla locus shows no evidence of transcript initiation from *NPEPPSP1* promoter. Instead, abundant transcription and isoforms are observed from MRPL45, and only three transcripts from testis could be identified that include non-deleted exons from *NPEPPSP1* and *TBC1D3*.

bioRxiv preprint doi: https://doi.org/10.64898/2026.01.14.699559; this version posted January 15, 2026. The copyright holder for this preprint (which was not certified by peer review) is the author/funder, who has granted bioRxiv a license to display the preprint in perpetuity. It is made available under aCC-BY 4.0 International license.

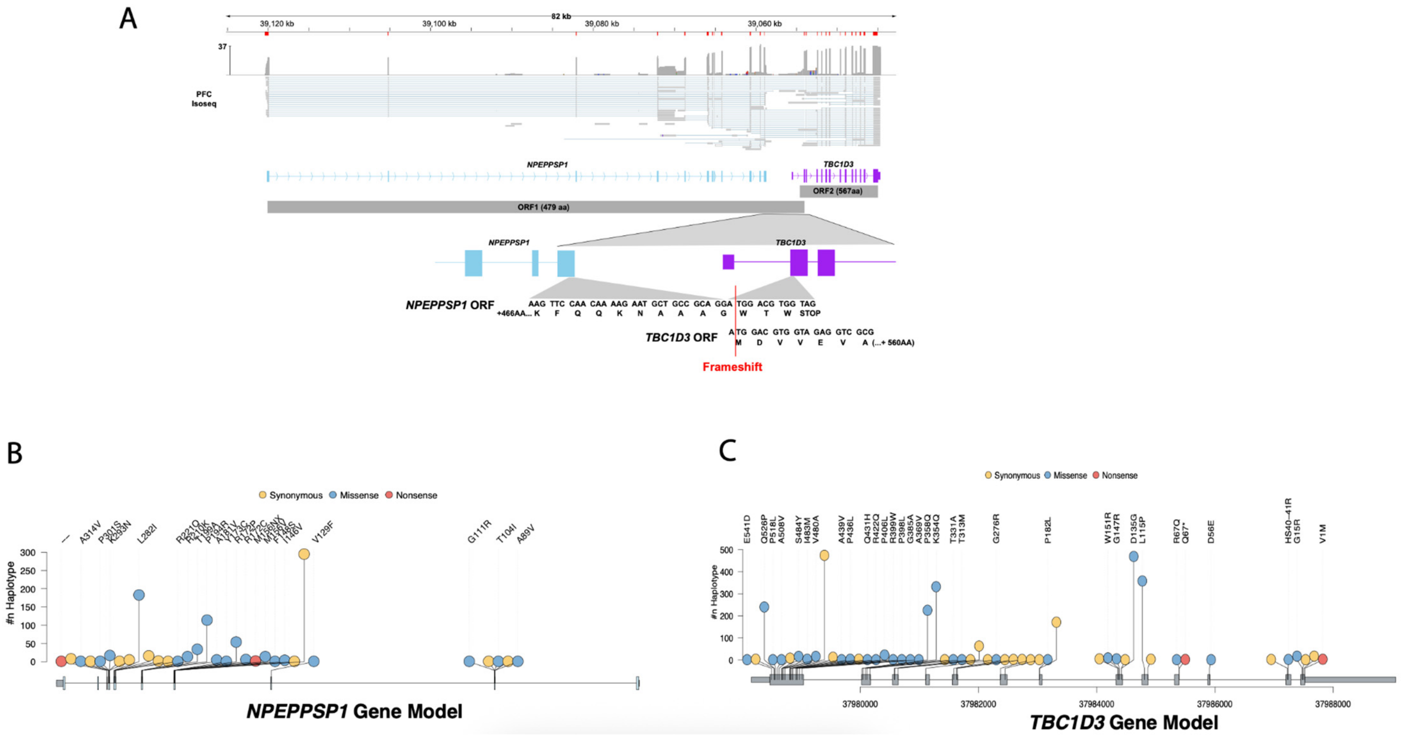


**Supplementary Figure 8. *NPEPPSP1-TBC1D3* retains two ORFs. A.** Iso-Seq data from the prefrontal cortex illustrates the fusion transcript across *NPEPPSP1* and *TBC1D3*. Below are two open reading frames (ORFs) identified with ORFfinder (Methods), which span *NPEPPSP1* and *TBC1D3* in separate reading frames overlapping by 13 nucleotides. **B.** Lollipop plots of *NPEPPSP1* illustrate synonymous (yellow), missense (blue), and nonsense (red) variants. Three missense variants were identified in 2,013 genome assemblies. **C.** Same as (B) but with *TBC1D3* ORF2. One missense variant was identified across the gene body.

bioRxiv preprint doi: https://doi.org/10.64898/2026.01.14.699559; this version posted January 15, 2026. The copyright holder for this preprint (which was not certified by peer review) is the author/funder, who has granted bioRxiv a license to display the preprint in perpetuity. It is made available under aCC-BY 4.0 International license.

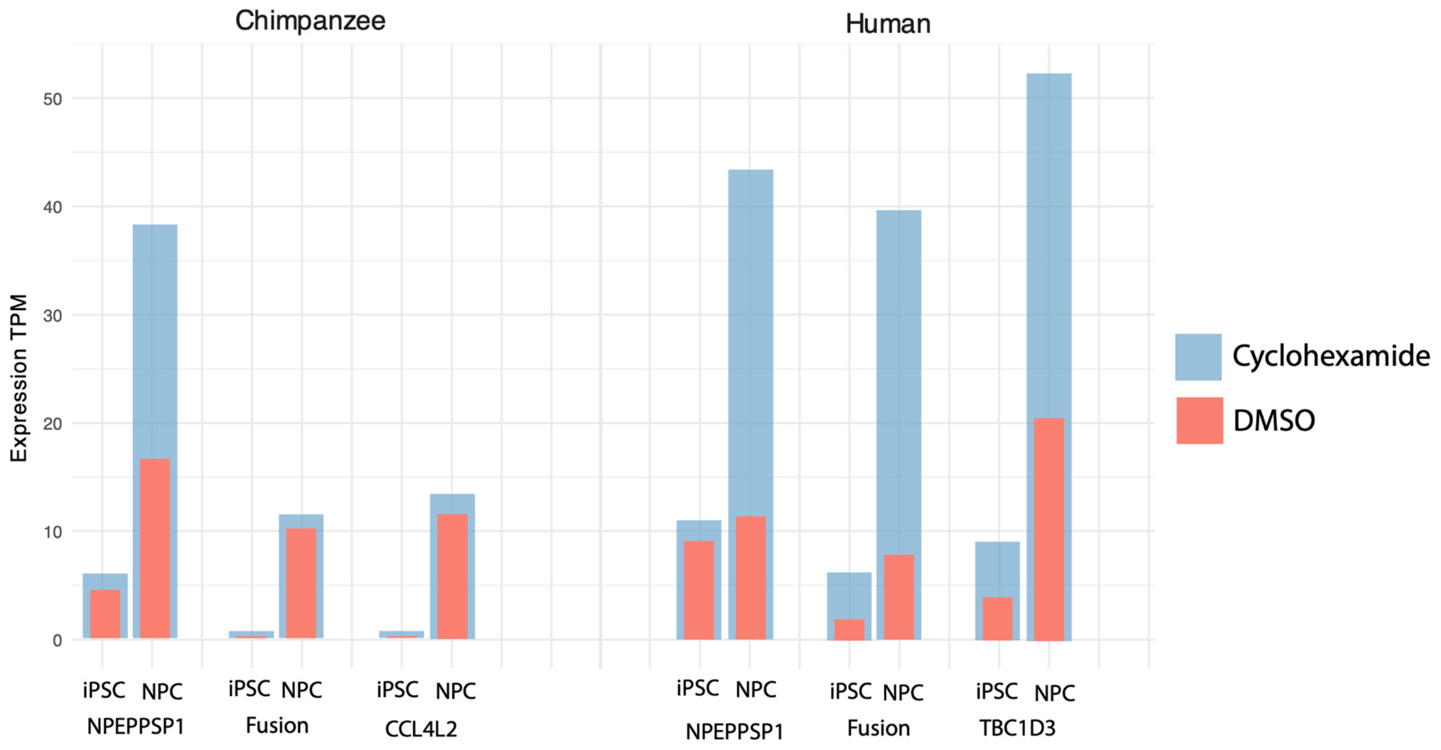


**Supplementary Figure 9. Disruption of nonsense mediated decay (NMD).** iPSCs and NPCs from chimpanzee and humans were treated with either DMSO or cycloheximide, a disruptor of NMD, to investigate posttranscriptional fate of fusion genes. Notably, *NPEPPSP1* is preferentially rescued in both species in NPCs relative to *TBC1D3*, though in humans both *NPEPPS1*, *TBC1D3*, and the fusion isoform increase equally.

bioRxiv preprint doi: https://doi.org/10.64898/2026.01.14.699559; this version posted January 15, 2026. The copyright holder for this preprint (which was not certified by peer review) is the author/funder, who has granted bioRxiv a license to display the preprint in perpetuity. It is made available under aCC-BY 4.0 International license.

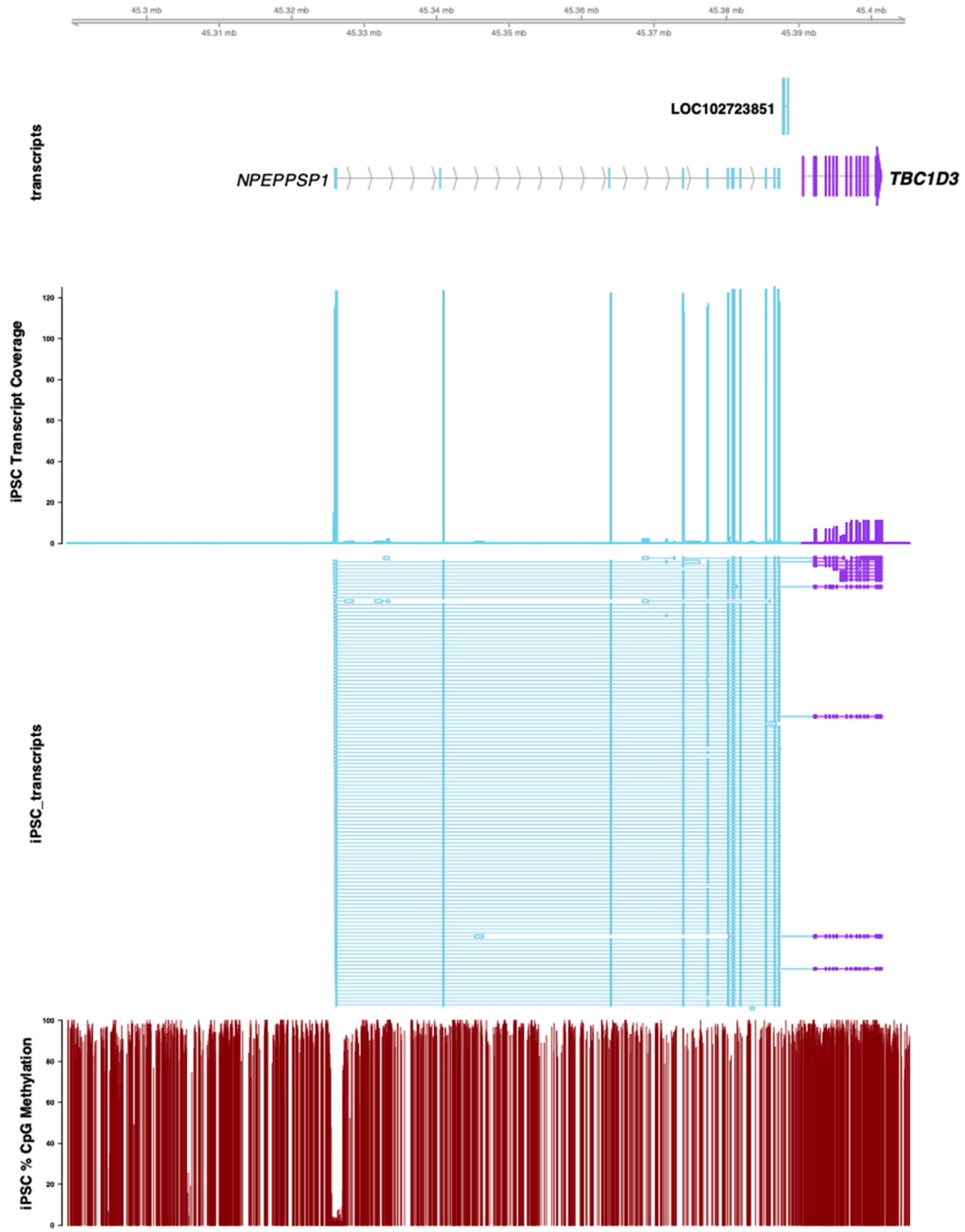


**Supplementary Figure 10. Human iPSC expression and methylation.** Human iPSC gDNA and full-length transcripts were mapped back to their donor-specific genome assembly (DSA) and annotated for *NPEPPSP1* (blue) or *TBC1D3* (purple) gene models. The *NPEPPSP1* promoter may be seen as a dip in methylation at *NPEPPSP1* exon 1.

bioRxiv preprint doi: https://doi.org/10.64898/2026.01.14.699559; this version posted January 15, 2026. The copyright holder for this preprint (which was not certified by peer review) is the author/funder, who has granted bioRxiv a license to display the preprint in perpetuity. It is made available under aCC-BY 4.0 International license.

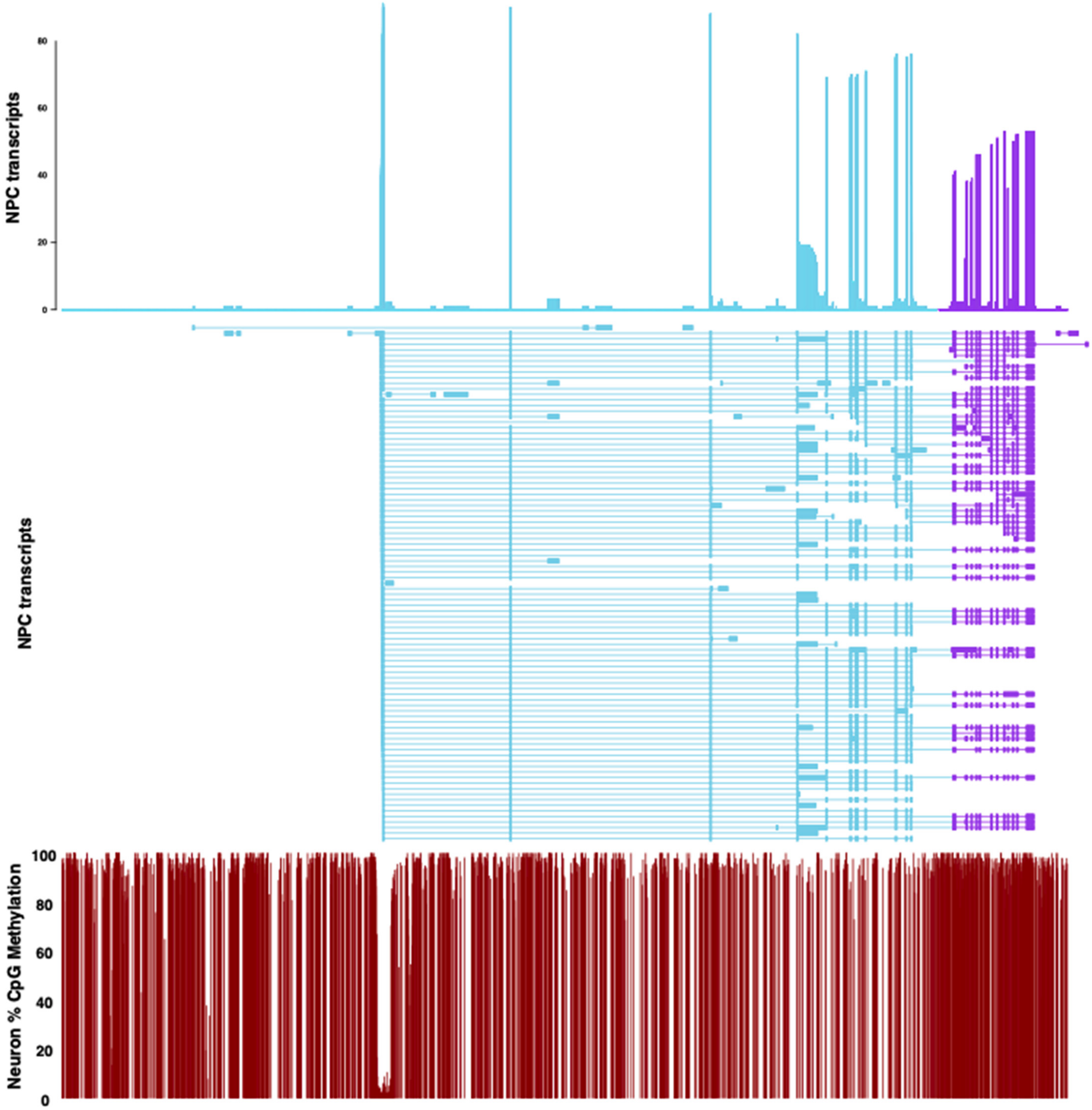


**Supplementary Figure 11. Human NPC expression and methylation.** Human NPC gDNA and full-length transcripts were mapped back to their DSA and annotated for *NPEPPSP1* (blue) or *TBC1D3* (purple) gene models. The *NPEPPSP1* promoter may be seen as a dip in methylation at *NPEPPSP1* exon 1.

bioRxiv preprint doi: https://doi.org/10.64898/2026.01.14.699559; this version posted January 15, 2026. The copyright holder for this preprint (which was not certified by peer review) is the author/funder, who has granted bioRxiv a license to display the preprint in perpetuity. It is made available under aCC-BY 4.0 International license.

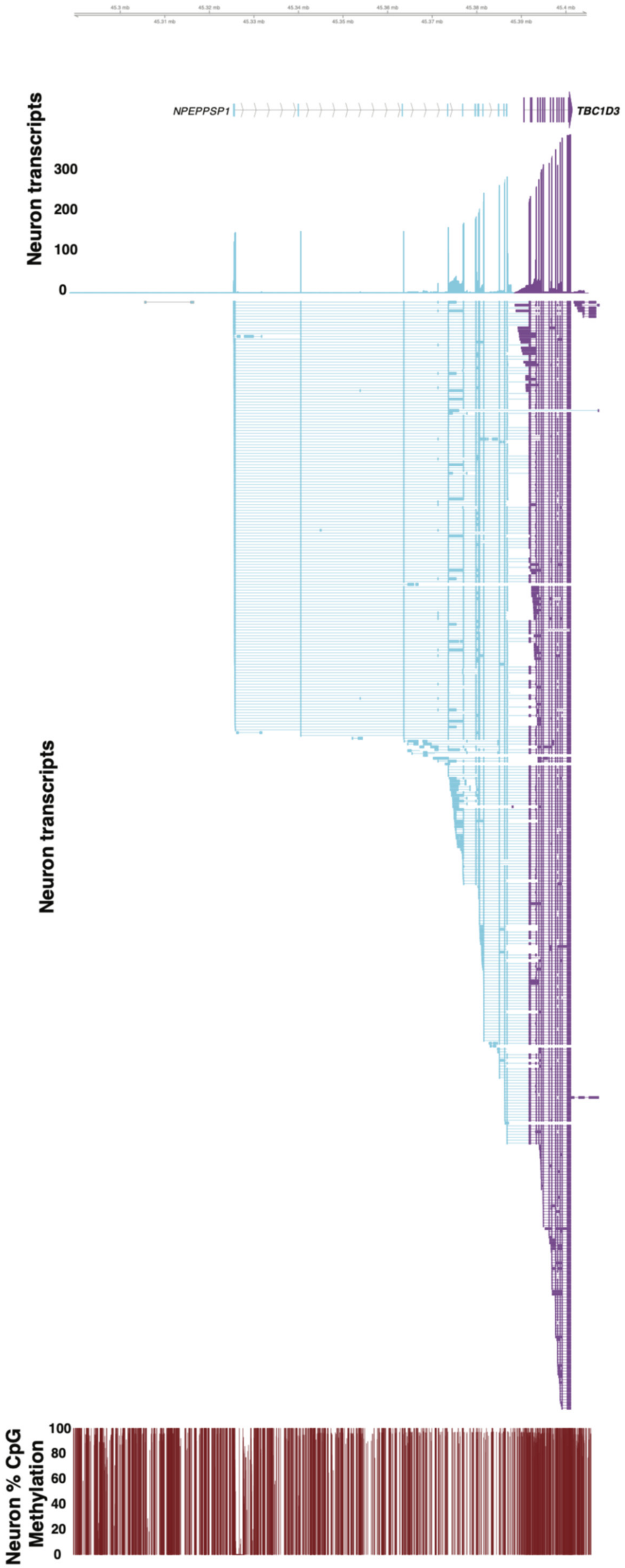

bioRxiv preprint doi: https://doi.org/10.64898/2026.01.14.699559; this version posted January 15, 2026. The copyright holder for this preprint (which was not certified by peer review) is the author/funder, who has granted bioRxiv a license to display the preprint in perpetuity. It is made available under aCC-BY 4.0 International license.

**Supplementary Figure 12. Human neuron expression and methylation.** Human neuron gDNA and full-length transcripts were mapped back to their DSA and annotated for *NPEPPSP1* (blue) or *TBC1D3* (purple) gene models. The *NPEPPSP1* promoter may be seen as a dip in methylation at *NPEPPSP1* exon 1. Notably, in neurons, *TBC1D3* has increased in expression relative to *NPEPPSP1*, in contrast to NPCs or iPSCs.

bioRxiv preprint doi: https://doi.org/10.64898/2026.01.14.699559; this version posted January 15, 2026. The copyright holder for this preprint (which was not certified by peer review) is the author/funder, who has granted bioRxiv a license to display the preprint in perpetuity. It is made available under aCC-BY 4.0 International license.

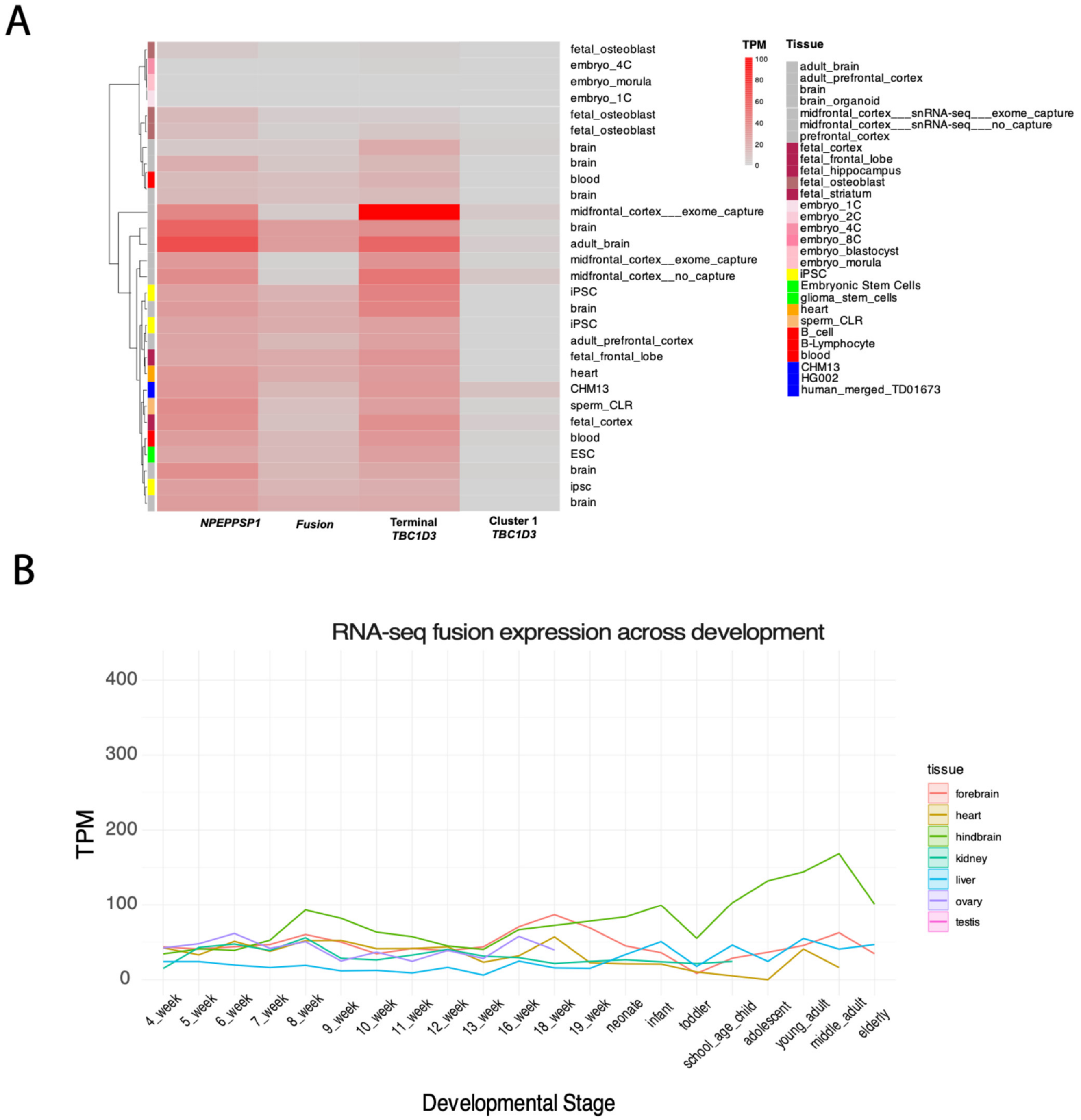


**<u>Supplementary Figure 13. *TBC1D3* expression.</u>** **A.** Full-length transcriptome sequencing of various libraries mapped to *NPEPPSP1*, *NPEPPSP1-TBC1D3* fusion, terminal *TBC1D3*, or any Cluster 1 *TBC1D3* (control). **B.** RNA-seq of *NPEPPSP1-TBC1D3* fusion across development.

bioRxiv preprint doi: https://doi.org/10.64898/2026.01.14.699559; this version posted January 15, 2026. The copyright holder for this preprint (which was not certified by peer review) is the author/funder, who has granted bioRxiv a license to display the preprint in perpetuity. It is made available under aCC-BY 4.0 International license.

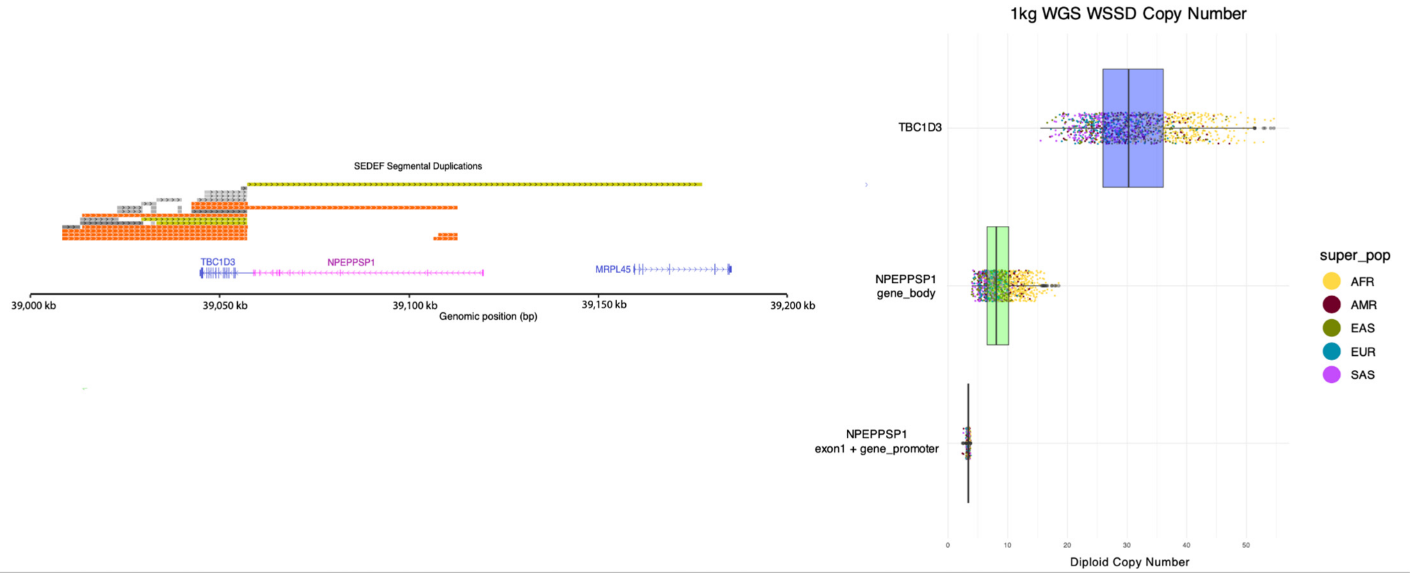


**<u>Supplementary Figure 14. *NPEPPSP1* promoter fixed in copy number.</u>** Left: Segmental duplication (SD) annotation of the *NPEPPSP1-TBC1D3* locus illustrate the numerous high-identity SDs across both *TBC1D3* and *NPEPPSP1* but does not include the *NPEPPSP1* exon 1 or its upstream promoter. Right: Diploid copy number estimates of *TBC1D3*, *NPEPPSP1*, and *NPEPPSP1* promoter in the 1000 Genomes Project (1KGP) identified by whole-genome shotgun sequence detection (WSSD; Sudmant et al., 2010) illustrate the fixed copy number of the *NPEPPSP1-TBC1D3* promoter relative to either *NPEPPSP1* or *TBC1D3*.

bioRxiv preprint doi: https://doi.org/10.64898/2026.01.14.699559; this version posted January 15, 2026. The copyright holder for this preprint (which was not certified by peer review) is the author/funder, who has granted bioRxiv a license to display the preprint in perpetuity. It is made available under aCC-BY 4.0 International license.

## SUPPLEMENTARY TABLES

### Supplementary Table 1: Neurospheres mappings by *TBC1D3* paralog

| TBC1D3 | Context | Promoter Identified | Neurospheres | Percentage of TBC1D3 Transcripts |
|---|---|---|---|---|
| 0 | NPEPPSP1 | yes | 51 | |
| 1 | Cluster2 (NPEPPSP1-fusion) | yes | 116 | 56.31% |
| 2 | Cluster2 | no | 0 | 0.00% |
| 3 | Cluster2 | no | 0 | 0.00% |
| 4 | Cluster2 | no | 11 | 5.34% |
| 5 | Cluster 2 | no | 2 | 0.97% |
| 6 | Cluster2 | no | 0 | 0.00% |
| 7 | Cluster2 | no | 0 | 0.00% |
| 8 | Cluster2 | no | 8 | 3.88% |
| 9 | Cluster 1 | no | 15 | 7.28% |
| 10 | Cluster 1 | no | 4 | 1.94% |
| 11 | Cluster 1 | no | 12 | 5.83% |
| 12 | Cluster 1 | no | 4 | 1.94% |
| 13 | Cluster 1 | no | 34 | 16.50% |
| | | | | |
| | | Total TBC1D3 Transcripts | 206 | |

bioRxiv preprint doi: https://doi.org/10.64898/2026.01.14.699559; this version posted January 15, 2026. The copyright holder for this preprint (which was not certified by peer review) is the author/funder, who has granted bioRxiv a license to display the preprint in perpetuity. It is made available under aCC-BY 4.0 International license.

### Supplementary Table 2: *NPEPPS* vs. *NPEPPSP1* mass spectra peptide observations

| ***NPEPPSP1* tryptic peptides** | **Datasets where peptide observed** | **Proteins with peptide** |
|---|---|---|
| MWLAAAAPSL | 23 | A6NEC2, P55786, E9PPD4, E7EWZ2…12 total |
| MWLAAAAPSLARRLLFL | 1 (breast cancer) | A6NEC2, P55786, E9PPD4…7 total |
| ARRLLFLGPP | 0 | 0 |
| PADVSPIN**C**S | 2 | A6NEC2 |
| FARTPV**T**STY | 3 | A6NEC2 |
| CV**C**VYTPVGK | 3 (Cytoplasmic Proteins of Untreated Cells) | A6NEC2 |
| AGAMENW**D**LV | 5 () | A6NEC2 |
| | | |
| ***NPEPPS* tryptic peptides** | **Datasets where peptide observed** | **Proteins with peptide** |
| MASFSMDCSP | MSV000079835 | E9PLK3, E9PJF9, E9PPZ2, |
| SFCVPGLWNP | MSV000083043, MSV000079835, MSV000080679, MSV000080826, MSV000080851, MSV000084248 | E9PLK3, E9PP11, E9PJF9, E9PPZ2, E9PP11, E9PLK3, E9PJF9, E9PPZ2 |
| PADVSPIN**Y**S | 117 | P55786, E9PLK3, E9PJF9, … (27 total) |
| FARTPV**M**STY | 7 | P55786, E9PLK3, P55786, …7 total |
| CV**R**VYTPVGK | 16 | P55786, E9PLK3, A0A7I2V389, …8 total |
| AGAMENW**G**LV | 22 | |

bioRxiv preprint doi: https://doi.org/10.64898/2026.01.14.699559; this version posted January 15, 2026. The copyright holder for this preprint (which was not certified by peer review) is the author/funder, who has granted bioRxiv a license to display the preprint in perpetuity. It is made available under aCC-BY 4.0 International license.

## Supplementary Table 3: iPSC-NPC-iNeuron transcript counts

| Species | Tissue | TOTAL_NPEPPS | NPEPPS | NPEPPSP1 | Total TBC1D3 | Terminal_TBC1D3 | CCL4L2 | Fusions | Total_Reads |
|---|---|---|---|---|---|---|---|---|---|
| Chimp | iPSC | 202 | 193 | 9 | 0 | 0 | 0 | 0 | 8489660 |
| Chimp | NPC | 88 | 75 | 89 | 1 | 0 | 50 | 46 | 8710214 |
| Chimp | neuron | 1214 | 559 | 655 | 8 | 1 | 511 | 454 | 21456687 |
| Human | iPSC | 172 | 115 | 57 | 10 | 3 | 0 | 1 | 10983570 |
| Human | NPC | 335 | 275 | 60 | 55 | 25 | 0 | 18 | 9156530 |
| Human | neuron | 937 | 767 | 170 | 217 | 208 | 0 | 98 | 22035016 |
| Orang | iPSC | 228 | 228 | 0 | 265 | 11 | 0 | 0 | 13648087 |
| Orang | NPC | 151 | 151 | 0 | 92 | 3 | 0 | 0 | 9127847 |
| Orang | neuron | 605 | 605 | 0 | 1125 | 11 | 0 | 0 | 21333770 |

## Supplementary Table 4: iPSC-NPC-iNeuron transcripts per million (TPM)

| Species | Tissue | NPEPPS_TPM | Total TBC1D3_TPM | Terminal_TBC1D3_TPM | CCL4L2_TPM | Fusion_TPM |
|---|---|---|---|---|---|---|
| Chimp | iPSC | 22.73 | 0 | 0 | 0 | 0 |
| Chimp | NPC | 8.61 | 0.1 | 0 | 5.7 | 5.3 |
| Chimp | neuron | 26.05 | 0.4 | 0 | 23.8 | 21.2 |
| Human | iPSC | 10.47 | 0.9 | 0.3 | 0 | 0.1 |
| Human | NPC | 30.03 | 6 | 2.7 | 0 | 2 |
| Human | neuron | 34.81 | 9.8 | 9.4 | 0 | 4.4 |
| Orang | iPSC | 16.71 | 19.4 | 0.8 | 0 | 0 |
| Orang | NPC | 16.54 | 10.1 | 0.3 | 0 | 0 |
| Orang | neuron | 28.36 | 52.7 | 0.5 | 0 | 0 |

bioRxiv preprint doi: https://doi.org/10.64898/2026.01.14.699559; this version posted January 15, 2026. The copyright holder for this preprint (which was not certified by peer review) is the author/funder, who has granted bioRxiv a license to display the preprint in perpetuity. It is made available under aCC-BY 4.0 International license.

## Supplementary Table 5: Human iPSC-NPC-iNeuron mappings by *TBC1D3* paralog

| TBC1D3 | Context | Promoter Identified | iPSC-DMSO | iPSC-CHX | NPC-DMSO | NPC-CHX | Neuron-DMSO |
|---|---|---|---|---|---|---|---|
| 0 | NPEPPSP1 | yes | 24 | 33 | 35 | 125 | 146 |
| 1 | Cluster2 (*NPEPPSP1* fusion) | yes | 10 | 23 | 60 | 142 | 196 |
| 2 | Cluster2 | no | 0 | 0 | 0 | 0 | 0 |
| 3 | Cluster2 | no | 0 | 0 | 0 | 0 | 0 |
| 4 | Cluster2 | no | 0 | 0 | 0 | 0 | 0 |
| 5 | Cluster2 | no | 0 | 0 | 0 | 0 | 1 |
| 6 | Cluster2 | no | 0 | 0 | 0 | 0 | 0 |
| 7 | Cluster2 | no | 0 | 0 | 0 | 0 | 0 |
| 8 | Cluster 1 | no | 0 | 0 | 0 | 0 | 0 |
| 9 | Cluster 1 | no | 0 | 0 | 0 | 0 | 0 |
| 10 | Cluster 1 | no | 0 | 0 | 0 | 0 | 0 |
| 11 | Cluster 1 | no | 0 | 0 | 0 | 0 | 0 |
